# Structural Dynamics and Optical Spectra of the Phycobiliprotein PC612 Complex

**DOI:** 10.1021/acs.jpcb.6c03535

**Published:** 2026-09-10

**Authors:** Dongyu Lyu, Sayan Maity, Ulrich Kleinekathöfer

**Affiliations:** † School of Science, 84498 Constructor University, Campus Ring 1, 28759 Bremen, Germany; ‡ Department of Physics and Astronomy and Thomas Young Centre, University College London, London WC1E 6BT, U.K.

## Abstract

Phycocyanin 612 (PC612) is a light-harvesting complex found in cryptophyte algae, characterized by its distinct open structure and flexible protein conformation, allowing the algae to survive in low-light aqueous environments. Although cryptophyte algae are known to show a diverse range of spectroscopic properties, the complex electronic structures and photophysical properties of their pigment molecules, known as bilins, are not yet fully understood, particularly in terms of how the surrounding protein environment influences them. In this study, we performed quantum mechanics/molecular mechanics simulations with excited-state calculations utilizing a numerically efficient density functional tight-binding method to examine the structural dynamics and optical features of bilin molecules within an open-form PC612 complex. Our results indicate that the protein matrix significantly controls the fluctuations of excitation energies and excitonic couplings of the pigment network. Very different from (bacterio)­chlorophyll-containing systems, thermal fluctuations induced by the environment have a minimal effect on the pigment spectral densities due to similar electron density distributions in the ground and excited states. The computed absorption spectra, calculated at different temperatures, are in reasonable agreement with the experimental counterparts, accurately predicting the relative peak positions and confirming the validity of our findings.

## Graphical abstract

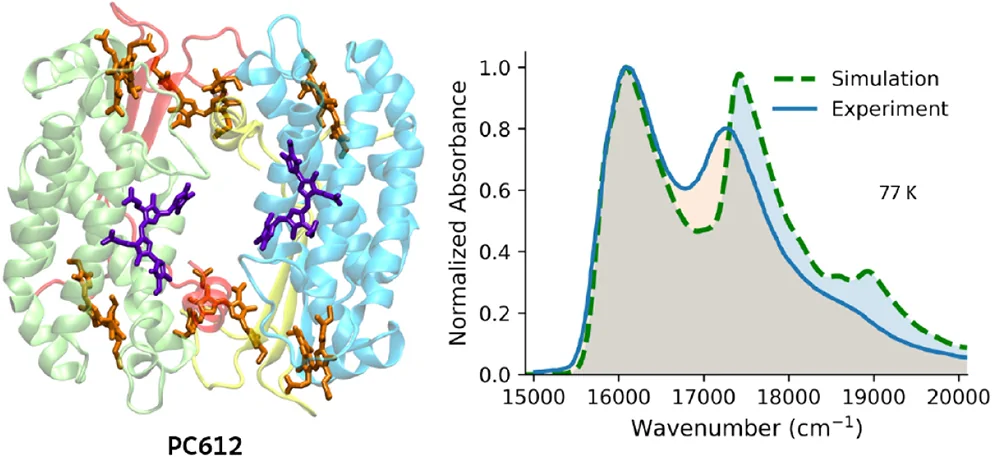

## Introduction

Photosynthetic antennae are pigment–protein complexes that specialize in harvesting sunlight and efficiently transferring the energy to the reaction center through a series of excitation energy transfer (EET) steps.^1–4^ Among different photosynthetic systems, cryptophyte algae provide a particularly interesting class of light-harvesting (LH) antennae complexes for studying EET processes. Cryptophytes are a group of small unicellular eukaryotic algae that inhabit marine and freshwater environments. They originated through secondary endosymbiosis, in which a heterotrophic eukaryotic host cell engulfed a red alga that had previously acquired a plastid from a cyanobacterium.^5,6^ This evolutionary history also resulted in a unique type of LH antenna complexes in cryptophytes, which are known as phycobiliproteins (PBPs), and they contain phycobilin pigments that are linear tetrapyrrole chromophores. Unlike chlorophyll molecules, these pigments are covalently attached to the water-soluble protein subunits.^6,7^ PBPs are also found in cyanobacteria and red algae, where they are typically organized into large phycobilisome complexes attached to the stromal surface of the thylakoid membrane. In cryptophytes, however, PBPs exhibit distinctive structural and organizational characteristics that differentiate them from those of other algae. During evolution, these antenna complexes underwent a structural reorganization from a trimeric form to a smaller heterodimeric assembly, accompanied by translocation into the thylakoid lumen.^8,9^ Consequently, unlike the membrane-associated phycobilisomes of cyanobacteria and red algae, cryptophyte biliproteins are present as a dense granular matrix filling the thylakoid.^10^

Due to the remarkably high excitation transfer efficiency of cryptophytes at low-light conditions at the bottom of shallow water, revealing the detailed mechanisms of the EET in these organisms has attracted significant interest.^11–13^ All PBPs share a similar structure, consisting of two highly similar β subunits and two more variable α subunits. Each α subunit carries one bilin chromophore, while each β subunit binds three bilins at fully conserved attachment positions.^14^ Depending on the bilin types embedded in the complexes, they are classified into two categories, phycocyanin (PC) and phycoerythrin (PE), exhibiting different absorption maxima.^6^ At the same time, the protein sequences are responsible for variations in the spectral properties of different PBPs as well.^14,15^ In particular, the insertion of a single aspartic acid residue in the α subunit of certain PBPs results in an approximately 73° rotation of the *αβ* protomers with respect to each other, producing a central cavity.^15^ This rotation leads to significant differences in the electronic couplings between the central bilins, due to variations in the pigment distances in the open and closed structures, e.g., the comparison between PC612 and PC645 is shown in Figure 1. The differences in the couplings significantly affect the spectral properties and energy transfer dynamics of these complexes.^15–19^

**Figure 1:**
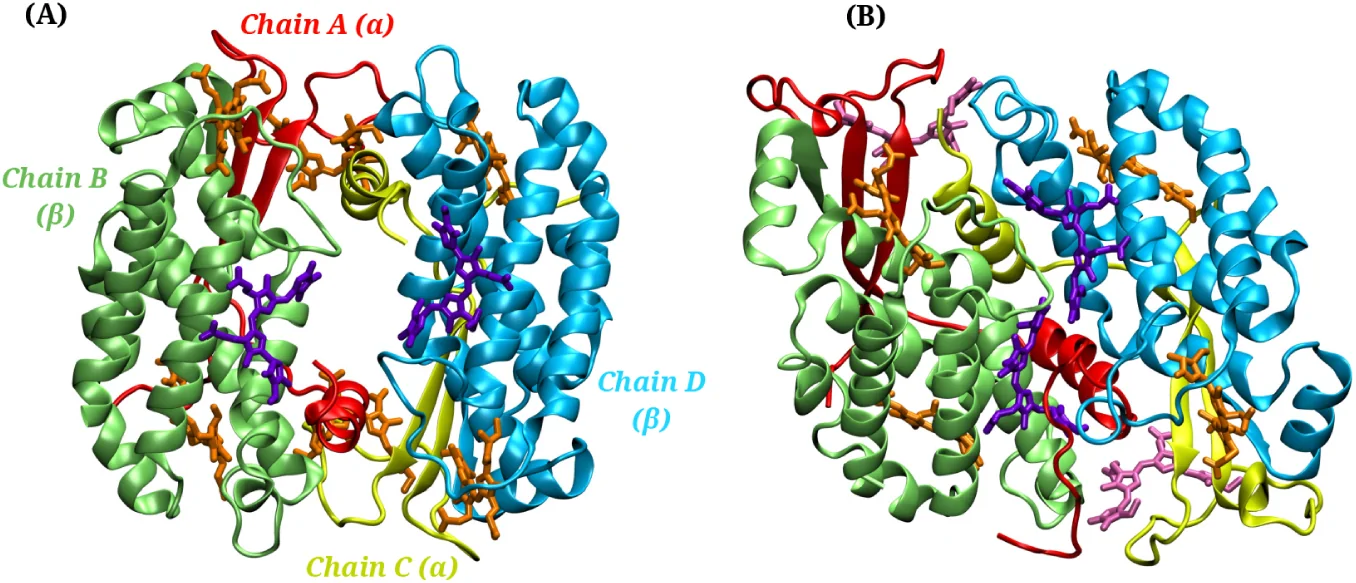
Comparison between (A) the open-form PC612 and (B) the closed-form PC645 complexes. The four peptide chains of the proteins are shown in different colors. PDB IDs: 4LM6 and 4LMS, respectively.^15^.

In the present study, we focus on the PC612 complex, which has an open-form structure, extracted from *Hemiselmis virescens* CCAC 1635.^15^ Figure 2A illustrates the structure of the complex, a homodimer composed of two subunits *αβ*, each carrying one dihydrobiliverdin (DBV) and three phycocyanobilins (PCBs) by covalent attachment. The chemical structures of the pigments DBV and PCB are shown in Figure 2B. The spatial organization of the subunits yields a quaternary structure with nearly 2-fold symmetry. Two distinct peaks can be seen in the experimental absorption spectrum, one at 575 nm, resulting from the contribution of the DBVs, and one at 612 nm originating from the PCBs.^15^ Previously, most experiments focused on the corresponding closed-form PC645 complex^20–23^ in which two PCBs in the α subunits are replaced by mesobiliverdins (MBVs). In PC645, the central DBV pigments are excitonically strongly coupled in contrast to the weakly coupled DBVs in the PC612 complex. This strong coupling between the DBV pigments in the PC645 complex leads to the formation of a delocalized molecular exciton pair, which plays a major role in facilitating efficient energy transfer in that complex.^23^ Moreover, ultrafast transient absorption experiments show that the energy transfer from the high-energy donor DBV pigments to the low-energy acceptor PCB molecules occurs in the open-form PC612 at twice the rate observed in the closed-form PC645 caused by the different coupling strengths within the pigment network.^24,25^

**Figure 2:**
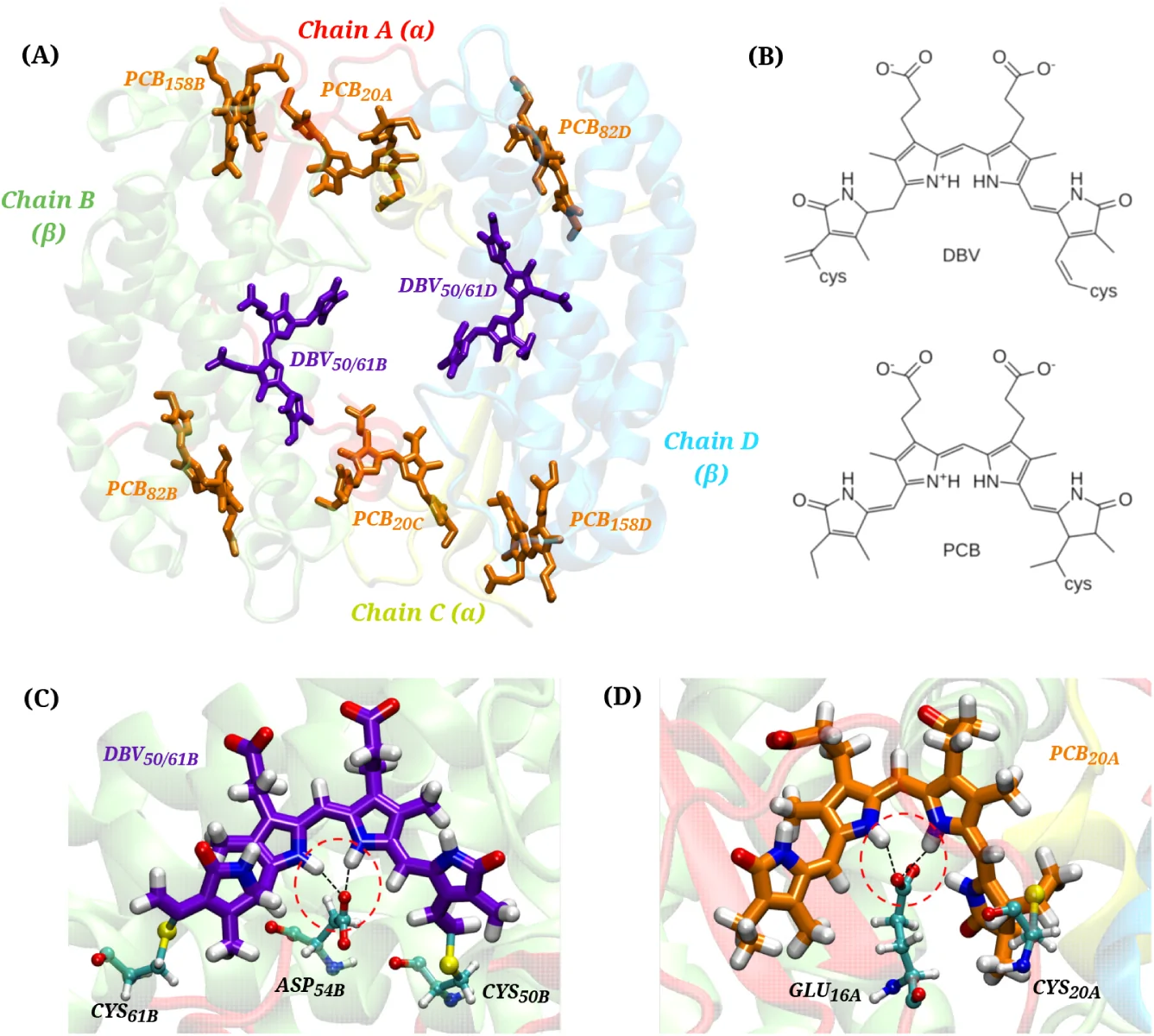
(A) The structure of the PC612 complex, which is an open-form phycobiliprotein containing eight bilins (two DBVs in purple and six PCBs in orange). The four peptide chains (chain A, B, C, and D) of the protein are colored in red, green, yellow, and cyan, respectively. (B) Chemical structures of the pigments present in the PC612 complex. (C, D) Example conformations of DBV and PCB pigments in the PC612 complex, indicating why these are assumed to be protonated at their central pyrrole rings due to interactions with nearby amino acid residues.

A series of theoretical studies have been performed to reveal the EET mechanism in open-form cryptophyte antennae complexes^18,19,26,27^ in particular by Curutchet and coworkers. The accurate determination of the excitation energies has been one of the challenges, requiring accurate quantum simulations and an efficient sampling strategy. They initially employed polarizable quantum/molecular mechanics (QM/MMPol) calculations, and the water solvent is described using the ddCOSMO continuum model. The excited state calculations were based on quantum/molecular mechanics (QM/MM) optimized geometries and the corresponding absorption spectra were computed afterward.^19^ This approach did, however, not fully resolve the two distinct absorption peaks. Subsequently, the sampling protocol was extended by extracting multiple snapshots from a classical molecular dynamics (MD) trajectory, followed by individual QM/MM optimizations and QM/MM MD simulations at the density functional theory (DFT) level.^27^ The full cumulant expansion (FCE) formalism^28,29^ was then applied to calculate the absorption spectra. Compared to pervious results, this refined protocol led to a significant improvement. The simulated spectra reproduced two distinct peaks with relative intensities in excellent agreement with experiment. Nevertheless, the energy separation between the two peak positions is still overestimated, with a value of approximately 1450 cm^–1^ compared to the experimental value of around 1050 cm^–1^.

In the present work, we have studied the structural dynamics followed by the optical properties of the open-form PC612 complex using a combined approach of QM/MM MD followed by excited state calculations also within a QM/MM framework. Both the ground and excited state calculations were performed using the numerically efficient but still accurate semiempirical DFTB approach. For the excited states this refers to the time-dependent long-range corrected density functional tight binding (TD-LC-DFTB) level of theory.^30^ The combined scheme of a DFTB ground state simulation and TD-LC-DFTB excited state calculations has been shown to be rather accurate for photosynthetic antenna complexes of bacteria, plants, and diatoms.^31–36^ In addition, we have benchmarked the TD-LC-DFTB results against TD-LC-DFT calculations for QM/MM optimized geometries. In the studied PC612 complex, all bilins were fully protonated at the central pyrrole rings because of their strong electrostatic interaction with the nearby protein residues. A detailed description of the determination of the protonation states is provided in the SI. This is in contrast to the closed-form PC645 complex in which one of the bilin molecules can be protonated or deprotonated depending on the pH value of the environment.^23^ An example of the electrostatic interactions of DBV and PCB pigments in the PC612 complex is shown in the lower panels of Figure 2. The site energies, excitonic couplings, spectral densities, and transition dipole moments of the PC612 complex are reported and compared to previous results.^19,27^ It was found that the site energies and excitonic couplings are strongly influenced by the protein environment. The spectral density, which characterizes pigment-environment coupling in open quantum system theory and serves as a key parameter in modeling exciton dynamics and optical line shapes, is, however, only minimally affected by the thermal fluctuations from the protein surroundings different from complexes containing (bacterio-)­chlorophyll molecules. A detailed analysis revealed that internal pigment vibrational modes predominantly determine the overall shape of the spectral density, including its low-frequency components. These unique vibration-coupled excitation features of bilin molecules are different from what is seen for the chlorophyll pigments of bacteria^37^ and were also reported earlier for the PE545 complex.^16,17^ Finally, absorption spectra computed using the FCE formalism^28,29^ exhibit substantially improved agreement with the experimental data compared to the earlier results, reproducing the two main peaks observed at different temperatures with peak separations closer to the experimental values, thereby further supporting the validity of the computational approach.

## Computational Protocols

The initial structure of the PC612 complex used for the simulation was obtained from the high-resolution crystal structure from Harrop et al.^15^ (PDB ID: 4LM6). Considering the aqueous protein environment, we assumed that the carboxylic acid groups of the bilins were deprotonated, similar to earlier studies.^23,38^ Furthermore, the nitrogen atoms of the central pyrrole rings are protonated. They form hydrogen bonds with nearby aspartate or glutamate residues in the protein, contributing to the electrostatic stabilization within the protein matrix (see Figure 2C and D).

The Ligand Reader & Modeler module from CHARMM-GUI Web server^39–41^ was utilized to customize the protonation state of the pigments. The amino acid residues were all assigned their standard protonation states as provided by CHARMM-GUI. Moreover, we have considered the GAFF2 force field for the bilin pigments as also present inside the CHARMM-GUI server. After adding hydrogen atoms, the complex was solvated using TIP3P water molecules within a 10.3 nm cubic box and then neutralized. After generating the initial topology and coordinate files from the CHARMM-GUI Web server, the ACPYPE (AnteChamber PYthon Parser interfacE) tool^42^ was employed to convert them into the GROMACS-format files for the simulation system. Furthermore, the bilin molecules in the system are all covalently linked to the sulfur atoms on the cysteine residues. The corresponding bond and angle parameters for the covalent linkage were added manually to the topology file using the same parameters as for the cysteine residue. Thereafter, a classical MD equilibration of the PC612 complex was carried out employing the GROMACS MD engine^43^ together with the AMBER 14SB force field.^44^ After energy minimization, a 2 ns NVT equilibration was carried out at 300 K, with a 1 fs integration time step to heat the system. This was followed by four consecutive 5 ns NPT equilibration steps, each utilizing a 1 fs time step. Position restraints were progressively lifted from to heavier atoms of various components of the system during these equilibration stages: initially both the protein and pigment molecules were constrained, followed by restraints on the protein alone, then on the protein backbones, and finally only on the C_α_ atoms of the protein. Afterward, a 10 ns NPT equilibration was conducted without any restraints, also using a 1 fs time step. To further stabilize the system, another 500 ns NPT equilibration was performed with a 2 fs integration time step. Hence, a total of 530 ns classical NPT equilibration was performed for the PC612 complex. The Nose-Hoover thermostat^45^ and Parrinello–Rahman barostat^46^ were employed to control the temperature and pressure, respectively. The short-range nonbonded interactions were set to 1.2 nm, and long-range electrostatic interactions were treated by the Particle Mesh Ewald (PME) method.^47^ In addition, the LINCS algorithm^48^ was used for keeping bond constraints, and as usual periodic boundary conditions (PBC) were applied throughout all simulations.

The final structure of the equilibration was used for the subsequent QM/MM dynamics. Due to a restriction in the present software implementation (see below), each pigment was handled as a single QM region in an individual simulation implicitly assuming uncorrelated fluctuations of the chromophores.^49,50^ Thus, each of the bilin molecules was assigned to a QM region in a separate simulation by introducing a cut at the C–S bond between the QM and the MM regions. The QM boundary was smoothed using hydrogen atom capping, while the partial charge of the terminal sulfur atom in the MM region was redistributed to its nearest neighboring atom. The rest of the system, containing water, ions, protein, and the other seven bilins, was treated as the MM region. For the QM region, the SCC-DFTB3 method^51^ with the 3OB-f parameter set^52^ was used, which describes vibrational frequencies of the C=C, C=N, and C=O bond stretching modes more accurately than the 3OB parameter set which is optimized for energetics. Following a 100 ps QM/MM NPT equilibration, a 40 ps QM/MM MD simulation with an integration time step of 0.5 fs was performed. The atomic coordinates were stored every 1 fs time extracting 40,000 frames. The simulations were performed using the GROMACS/DFTB+ interface^53,54^ and the extracted geometries were subsequently used to calculate the spectral densities. In addition, an extended simulation of 1 ns was carried out with a time step of 1 fs starting at the end of the 100 ps NPT equilibration. The coordinates for this run were saved every 100 fs, resulting in 10,000 frames. The resulting trajectories from all these QM/MM ground state simulations were subjected to excited state calculations employing the TD-LC-DFTB2 method,^30^ using the DFTB+ 21.1 package.^54^ Subsequently, for the simulations with the 1 fs time step the autocorrelation functions were determined from the site energy fluctuations, and the spectral densities were calculated by performing a cosine transformation.^31,55^ To improve sampling for the spectral densities, we performed the same QM/MM MD simulations followed by TD-LC-DFTB calculations for two other sets of initial geometries, each extracted from a 200 ns classical MD trajectory at 100 ns intervals. In addition, after 1 ns of QM/MM MD, each system was energy-minimized using DFTB/MM to obtain optimized structures. These geometries were subsequently used for TD-DFT calculations employing the CAM-B3LYP functional with the Def2-TZVP basis set within the Tamm-Dancoff approximation (TDA) as implemented in the ORCA quantum chemistry program,^200^ as well as for TD-LC-DFTB calculations, in order to assess the accuracy of the TD-LC-DFTB method for the studied bilin molecules. Furthermore, the TD-DFT results obtained from individually QM/MM optimized structures were analyzed using Multiwfn^56^ to derive atomic transition charges from electrostatic potentials (TrEsp) method.^57,58^ The same procedure was applied to the bilins in the closed-form PC645 complex recently,^23^ and these TrESP charges were further employed in the calculation of excitonic couplings. To be able to calculate the excitonic couplings between the pigments, the previously performed 500 ns classical MD simulation was extended by an additional 1 μs. From the resulting trajectory, 100,000 snapshots were extracted for the excitonic coupling calculations. To ensure sufficient conformational sampling, two additional independent 1.5 μs classical MD simulations were carried out. For each replicate, snapshots were extracted from the last 1 μs following the same protocol, and the excitonic couplings were calculated accordingly. An summary of the trajectories used in this study is provided in the SI.

## Results and Discussion

### Structural Flexibility and Symmetry

To characterize the structural flexibility of the PC612 complex, we calculated the root-mean-square fluctuation (RMSF) of the backbone atoms from the first 1 μs classical MD trajectory, as shown in Figure 3. The asterisks indicate the positions of cysteine residues covalently linked to the corresponding bilins. Although some individual residues show noticeable differences in fluctuation amplitudes, the RMSF profiles of the structurally equivalent chains (A and C, B and D) are quite consistent. At the same time, it already becomes evident that a trajectory of 1 μs is not sufficient to obtain proper sampling of all conformations since otherwise the symmetry should become even more evident in the RMSF plot. Figure 3 reveals that the loop regions of the protein generally exhibit higher flexibility than the more structured α-helices and β-sheets. In particular, the loops comprising residues 15–35 in chains A and C, corresponding to continuous residue indices 15–35 and 254–274 in Figure 3, show enhanced flexibility. This region is directly involved in the covalent attachment and interactions with PCB_20_. Its enhanced flexibility may contribute to increased conformational fluctuations of the bilin molecules as well. The loop regions in chains B and D also display a relatively high flexibility. However, the corresponding bilins are not located within these flexible regions, and their interactions with these loops are relatively limited. Therefore, these flexible regions are expected to have a smaller impact on the conformational dynamics of the associated bilins.

**Figure 3:**
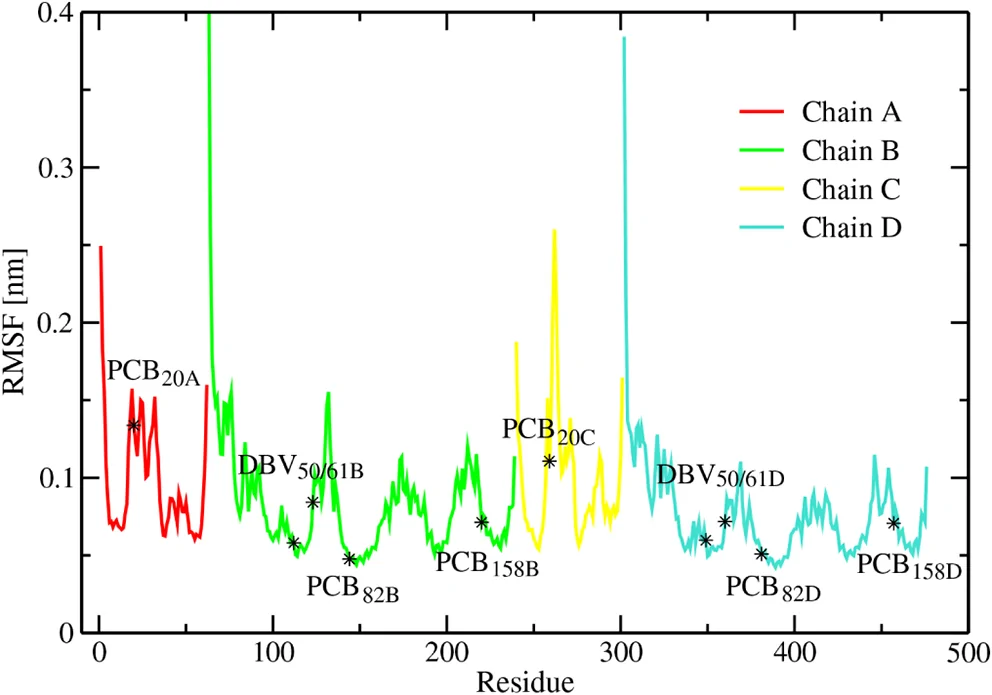
RMSF of backbone atoms calculated based on a 1 μs classical MD trajectory of the PC612 complex. The asterisks indicate the positions of the cysteine residues covalently linked to the corresponding bilin molecules in the protein sequence. For clarity, the residues are numbered consecutively across the four chains.

In total, three independent 1.5 μs classical MD simulations were performed. In all three simulations, PCB_20A_ showed a significant conformational change. In the initial crystal structure, the nitrogen atoms of rings A and D are located on opposite sides of the linear tetrapyrrole molecule formed by rings B and C, as illustrated in the left panel of Figure 4. During the simulations, however, the nitrogen of ring A undergoes a flip toward the carboxyl group of residue Glu16A. As a result, the nitrogen atoms of rings A, B, and C are on the same side of the molecule, while the one of ring D remains on the opposite side, as shown in the right panel of Figure 4. These two conformations were observed to interconvert repeatedly throughout the simulations. The pronounced conformational flexibility of PCB_20A_ can be attributed to its location at the periphery of the complex. In particular, the bilin is exposed to the solvent and ring A is not stabilized by strong interactions with nearby amino acid residues, allowing it to adopt multiple conformations with relative ease. Despite the structural symmetry of the complex, however, this conformational change occurred only for PCB_20A_. Its symmetry-related counterpart, PCB_20*C*_ on chain C, retained a conformation similar to the one found in the crystal structure throughout all simulations without undergoing any comparable conformational transition. This finding may originate from subtle asymmetries in the initial crystal structure. Although the amino acid sequences of chains A and C are identical, the local conformations of the loop regions accommodating the bilins are not completely equivalent. These small structural differences in the starting configuration may prevent the two symmetry-related sites from exhibiting identical behavior within the limited time scale of the simulations. It is likely that PCB_20*C*_ undergoes a similar conformational transition in extended simulations but initially seems locked in a rather stable conformation.

**Figure 4:**
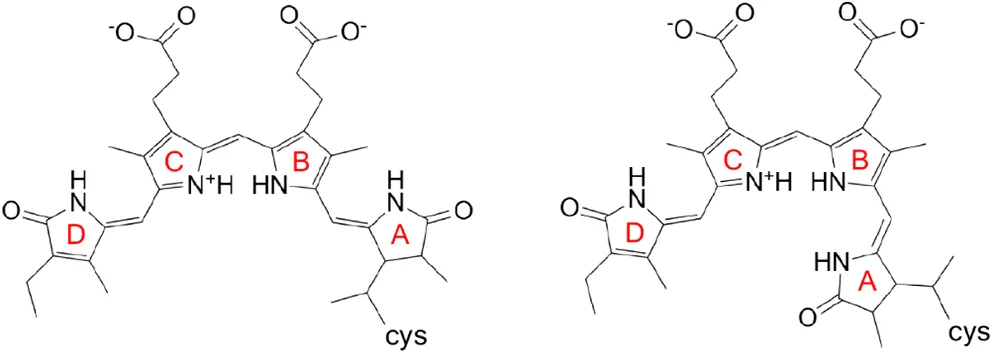
Two possible conformations of PCB_20A_ observed during classical MD simulations.

The structure obtained after 500 ns of the first classical MD simulation was selected as the starting structure for the subsequent QM/MM MD simulations and excited-state calculations. In this structure, PCB_20A_ and PCB_20*C*_ happened to adopt two different conformations. In the following sections, we would demonstrate that this conformational difference does not significantly affect the excited-state properties or the calculated absorption spectra.

### Impact of the Protein Matrix on the Site Energies

To study the exciton dynamics in LH complexes within the Frenkel model, the excitonic Hamiltonian of the system can be written as^59^

1 $$H=\underset{m}{\sum }E_{m}\left|m\right\rangle \left\langle m\right|+\underset{m\neq n}{\sum }V_{mn}\left|n\right\rangle \left\langle m\right|$$

where the diagonal elements *E*_*m*_ are the excitation energies, known also as site energies, and the off-diagonal elements *V*_*mn*_ represent the respective couplings. This Hamiltonian is defined within the single-exciton manifold, thus, de-excitations to the electronic ground state, as well as double (and higher) excitations, are not included in this model. The excitation energies of the pigments to the lowest excited state, i.e., the energy gap between the S_1_ and the ground states, is a crucial quantity that has to be be extracted taking the protein environment into account. Therefore, we have adopted a QM/MM approach, as outlined in the section **Computational Protocols**. Since the trajectory analysis is based on the semiempirical TD-LC-DFTB method and the excited state electronic structure of bilin pigments is complex, we first benchmarked this method against the time-dependent long-range corrected DFT (TD-LC-DFT) approach. In order to do so, we performed TD-LC-DFT calculations (CAM-B3LYP/Def2-TZVP) on QM/MM optimized structures to extract the lowest excitation energies of the different pigments. We then repeated the same calculations using the TD-LC-DFTB method. The comparison is shown in Figure 5, where the excitation energy profile of the PC612 complex is shown. The unshifted data is shown in Figure S4. The figure illustrates a similar trend in excitation energies for both the TD-LC-DFT and TD-LC-DFTB methods, suggesting that TD-LC-DFTB can be effectively applied for trajectory analysis across multiple frames. We want to mention that we are only interested in the relative energies between the pigments since the absolute energies will not influence the exciton dynamics, but instead lead only to an overall shift in spectra.^36^ Thus, a shift between the different energy profiles is of no concern here. To extract the excitation energies along trajectories, we considered both the QM/MM MD trajectories of 40 ps and 1 ns duration. To extract the lowest excitation energy along the trajectory can be challenging for bilin pigments because of the crossing of higher level excited states with the S_1_ state as already mentioned earlier.^16,17,60^ Thus, we here considered the optically most bright excitation between the five excited states to consider the correct state excited state along the trajectory. An example of the extracted state is shown in Figure S6 in the Supporting Information.

**Figure 5:**
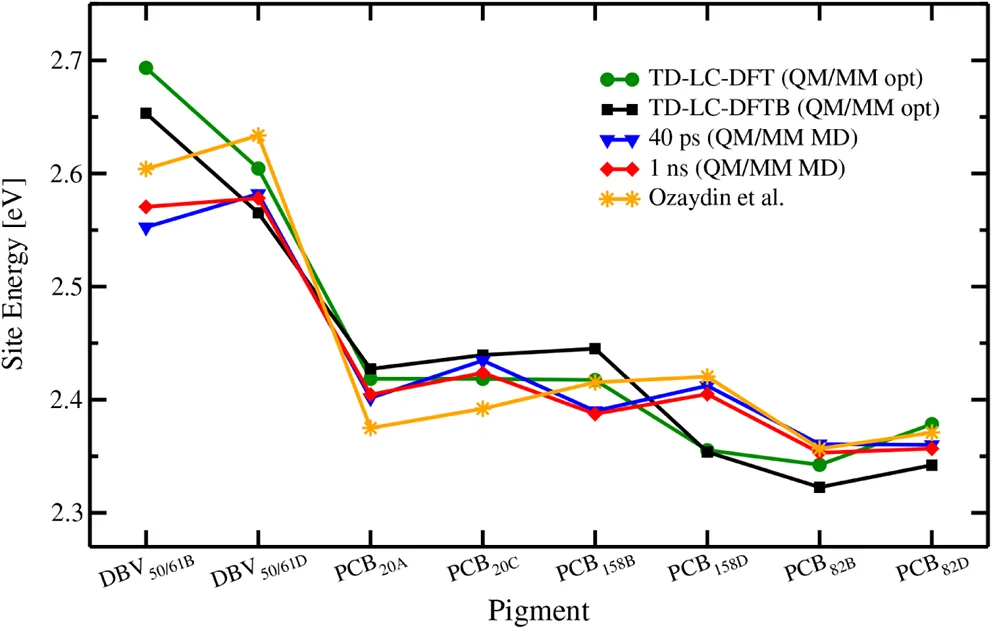
Site energies of the pigments in the PC612 complex, calculated using the TD-LC-DFT and TD-LC-DFTB methods based on the QM/MM optimized structures. Moreover, the average site energies based on TD-LC-DFTB calculations along the 1 ns and the 40 ps QM/MM MD trajectories are shown. Additionally, the data obtained from the QM/MM MD trajectories reported by Ozaydin and Curutchet^27^ is included for comparison. All curves have been shifted such that they have the same average site energies of the PCB molecules.

The average energies and their fluctuations are also depicted in Figure 5 while the averages obtained from the two trajectories are highly consistent. Moreover, the site energies of the symmetry-related pigments within the complex are nearly identical, despite the conformational differences described in the previous section. In both the QM/MM-optimized structures and the QM/MM MD trajectories, the DBVs, which are the two bilins located at the center of the complex, exhibit the highest site energies. They predominantly contribute to the highest-energy excitonic states in the PC612 complex, whereas the PCB_82_ pigments contribute most strongly to the lowest-energy excitonic states, as shown in Table S2 of the Supporting Information. This behavior is consistent with the energy transfer mechanism reported in previous studies for the closed-form PC645 complex under high-pH conditions.^11,20,23,38,61^ For comparison, the site energies obtained from QM/MMPol calculations at the TD-CAM-B3LYP/6-31G­(d) level of theory along QM/MM trajectories from Curutchet and co-workers^27^ are also shown in Figure 5. These results show a similar trend in the overall excitation energy ladder of the PC612 complex, especially when compared with the present findings obtained from our QM/MM trajectories. However, the relative energy gap between DBV and PCB is slightly larger in the QM/MMPol results, by approximately 0.05 eV.

The distributions of the excitation energies obtained along the 1 ns QM/MM MD simulation are shown in Figure S5 in Supporting Information. While all excitation energy distributions exhibit roughly Gaussian shapes, they vary in terms of peak positions, widths, and slight skewness. Furthermore, the system displays a notable degree of symmetry: regardless of the chromophore type, the bilins bound to the same residue site in chain B and in chain D (or chain A and C) exhibit not only quite similar site energies but also very comparable energy distributions.

### Excitonic Couplings and Excitonic Hamiltonian

The excitonic coupling is another key parameter in the system Hamiltonian shown in eq 1. In the present study, we have employed the TrEsp approach^57,58^ proposed by Renger and coworkers to calculate these couplings. The coupling expression is given by

2 $$V_{mn}=\frac{f\left(R_{mn}\right)}{4\pi \epsilon_{0}}\underset{I\in m}{\sum }\underset{J\in n}{\sum }\frac{q_{I}^{T}\cdot q_{J}^{T}}{\left|r_{m}^{I}-r_{n}^{J}\right|}$$

where $q_{I}^{T}$ and $q_{J}^{T}$ denote the atomistic transition charges of atoms *I* and *J*, *R*_*mn*_ is the distance between the centers of bilins *m* and *n*, and *f*(*R*_*mn*_) is a distance-dependent screening factor taking environmental influences on the excitonic coupling into account. Both a constant screening factor *f* = 0.69 and an exponential form derived by Curutchet et al.^62^ have been considered. The exponential one can be written as

3 $$f\left(R_{mn}\right)=A\exp\left(-BR_{mn}+f_{0}\right)$$

where the values of parameters *A*, *B*, and *f*_0_ are 2.68, 0.27, and 0.54, respectively.^62^

As mentioned in the section Computational Protocols, 100,000 frames that have been extracted from the 1 μs classical MD simulation to determine the average excitonic coupling values. Only average couplings are determined since with the restriction of our current code which can only treat one QM region at a time, no couplings can be determined at the same time as the site energies. This approximation, however, is minor since replacing the time-dependent coupling values by time-averaged ones has only a minor influence on the exciton dynamics.^60^

Following the calculation of the excitonic couplings, their values have been combined with the average excitation energies obtained along the 1 ns QM/MM MD trajectory to yield the excitonic Hamiltonian of the PC612 complex. The resulting Hamiltonian entries are listed in Table 1 based on the constant and the exponential screen factors. The average excitonic Hamiltonian, constructed using the coupling values calculated with the constant screening approach, was then employed in modeling the absorption spectra, as discussed below.

**Table 1:** Hamiltonian of the PC612 Complex^a^

Bilins	DBV50/61*B*	DBV50/61*D*	PCB_20A_	PCB_20*C*_	PCB_158*B*_	PCB_158*D*_	PCB_82*B*_	PCB_82*D*_
DBV50/61*B*	20,732	24	20	–9	33	–1	–62	20
DBV50/61*D*	19	20,794	–9	19	–3	35	21	–63
PCB_20*A*_	16	–7	19,394	–7	82	3	17	31
PCB_20*C*_	–7	15	–5	19,548	2	117	29	18
PCB_158*B*_	26	–2	66	2	19,256	7	18	7
PCB_158*D*_	–1	27	2	94	5	19,397	8	18
PCB_82*B*_	–49	16	13	23	14	6	18,979	5
PCB_82*D*_	16	–50	25	14	6	14	4	19,008

a The site energies given on the diagonal have been extracted from the 1 ns QM/MM MD trajectories. The average excitonic couplings with the constant screening factor and the exponential form are tabulated in the upper and lower triangles , respectively. All energy values are given in units of cm^–1^.

Due to the rotation of the *αβ* subunit, the distance between the two central DBVs in the open-form PC612 complex is significantly larger than in the closed-form PC645 complex. Consequently, the corresponding excitonic coupling is reduced to 24 cm^–1^, compared to 477 cm^–1^ in the closed-form PC645 complex.^23^ The strongest excitonic coupling values in the PC612 complex can be seen between PCB_20*C*_ and PCB_158*D*_, as well as between their symmetric counterparts, PCB_20*A*_ and PCB_158*B*_, with magnitudes of 117 cm^–1^ and 82 cm^–1^, respectively.

The distributions of the seven strongest excitonic coupling values in the PC612 complex including the constant screening factor are depicted in Figure 6. Although the excitation energies of the equivalent bilin pairs in chains B and D (or chains A and C) exhibit a high degree of symmetry, the peak positions and widths of the corresponding excitonic coupling distributions differ significantly for two pair for which one would have expected also quite similar results. In particular, the two possible conformations adopted by PCB_20*A*_ during the simulations lead to differences in the corresponding excitonic couplings compared to PCB_20*C*_. The distributions of excitonic couplings obtained from the three independent simulations are presented in Figure S7 of the Supporting Information. Although the coupling distributions exhibit significant differences, the averaged coupling values are more consistent among the different simulations. For example, the coupling between PCB__20*A*__ and PCB_158*B*_, which shows the largest variation in its distribution, has average values of 82, 88, and 92 cm^–1^ in the three simulations, respectively. Moreover, the absorption spectra calculated using these different coupling data sets show negligible differences, as demonstrated in Figure S11.

**Figure 6:**
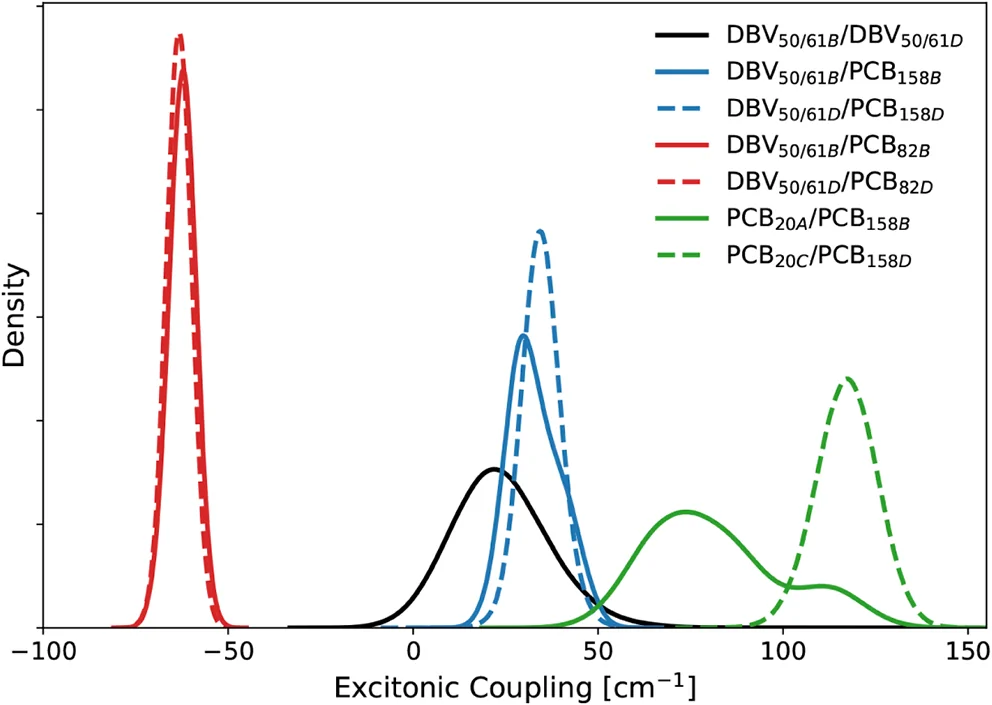
Distributions of the strong excitonic couplings using a constant screening factor.

### Effect of Environmental Fluctuations and Spectral Densities

Spectral densities characterize how the primary modes of interest, here the electronic excitations, couple to vibrational modes of the environment but also of the pigments themselves. They serve as a crucial input in open quantum system models of light-harvesting complexes.^63^ Different theoretical or experimental methods can be employed to determine spectral densities. In the present study, the spectral density for pigment *m* is calculated from excitation energy fluctuations of that pigment along QM/MM molecular dynamics trajectories employing the TD-LC-DFTB approach. This method has been tested on LH complexes from bacteria,^31^ plants^32–35^ and diatom algae^36^ resulting in good agreement with experimental outcomes. The spectral density for pigment *m* can be determined as

4 $$J_{m}\left(\omega \right)=\frac{\beta \omega }{\pi }\int_{0}^{\infty }dtC_{m}\left(t\right)\cos\left(\omega t\right)$$

where β = 1/*k*_*B*_ *T* denotes the inverse temperature. The site energy autocorrelation function *C*_*m*_(*t*) can be determined from a time series of the energy gaps as

5 $$C_{m}\left(t_{l}\right)=\frac{1}{N-l}\overset{N-l}{\underset{k=1}{\sum }}\Delta E_{m}\left(t_{l}+t_{k}\right)\Delta E_{m}\left(t_{k}\right)$$

where *E*_*m*_ is the site energy of the pigment *m* and Δ*E*_*m*_ = *E*_*m*_ – ⟨*E*_*m*_⟩. Moreover, *N* denotes the number of snapshots for a particular trajectory window, and *l* is the index of the time shift *t*_*l*_ in the autocorrelation function.

Results for the spectral densities of the eight pigments are shown in Figure 7. This figure shows a view restricted to the low-frequency region up to 800 cm^–1^ while the spectral densities for the full relevant frequency range are depicted in Figure S8 in the Supporting Information. For the same type of bilins, the spectral densities of the individual pigment are similar despite differences in their local protein environments. In contrast, bilins with different chemical structures, i.e., DBV and PCB, exhibit differences in their spectral densities, particularly in the region around 1200 cm^–1^. Moreover, although the peak positions are consistent with the spectral densities reported by the Curutchet group,^19^ the amplitudes obtained in the present work are noticeably larger. In particular, the peak near 800 cm^–1^ exceeds 1500 cm^–1^ in our calculations, whereas the corresponding feature in the work of Curutchet and coworkers remains below 1000 cm^–1^. These differences are most likely due to the different approaches used to compute the excited-state properties, with the present work employing TD-LC-DFTB instead of TD-DFT.

**Figure 7:**
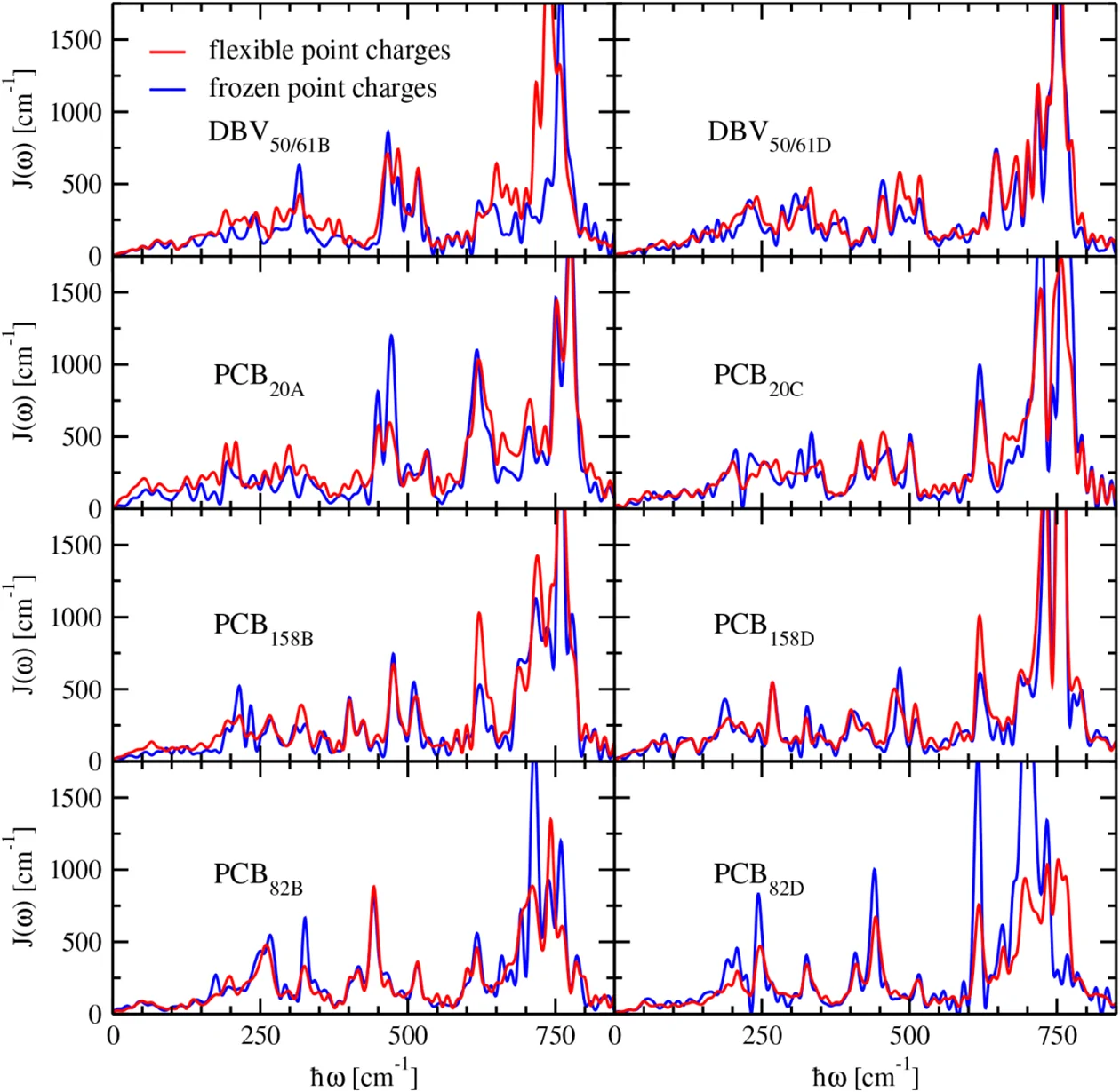
Low-frequency region of the spectral densities for the eight individual bilin molecules in the PC612 complex. Shown are two variants based on the same trajectories, one with full QM/MM coupling (moving point charges) and the other with frozen environmental point charges.

In the next step, we tested how thermal fluctuations of the pigments and the protein environment affect the spectral densities. In order to do so, we repeated the excitation energy calculations using a frozen point-charge environment in the QM/MM framework and extracted the spectral densities from the resulting energy fluctuations. In the DFTB+ implementation, the MM point charges are incorporated as an external electrostatic field rather than as explicit particles interacting directly with the QM atoms. Therefore, the frozen environment treatment does not involve direct QM–MM overlap issue. Nevertheless, we monitored the distances between the QM bilin and the frozen MM environment along the trajectories to verify that no unphysical close contacts were introduced. For a bilin system, such an approach was already performed for the PE545 system.^16,17^ For (bacterio-)­chlrophyll systems the effect of the surrounding can be performed by simple neglecting the environmental charges, for bilin systems this, however, leads to many crossings between excited state energies along a trajectory which can be avoided by a static external field. The results for the test calculations are shown in Figure 7 and in Figure S8. These figures demonstrate that the spectral densities of the bilin pigments are qualitatively similar when the environmental point charges are kept fixed, with the peak positions remaining unchanged. This suggests that the same vibrational modes contribute to the electronic excitation, although variations in the peak amplitudes are observed. In standard QM/MM simulations, however, the external charges change their positions over time. This behavior of the spectral densities shows that by far the major contributions to the spectral densities in the bilin systems stem from internal vibrational modes of the bilins rather than from fluctuations in the surrounding protein or solvent. Also in (bacterio-)­chlorophyll systems internal modes do contribute to the low-frequency part of the spectral density^37^ but are, depending on the system, majorly influenced by fluctuations of the protein and water environment.

### Excitation Profile of Bilin Pigments within Protein

To characterize the excitation profile of the bilin pigments within the protein environment, we analyzed the Highest Occupied Molecular Orbital (HOMO), and Lowest Unoccupied Molecular Orbital (LUMO). As an example, Figure 8 displays the data for DBV_50/61*B*_, and Figure S10 in the SI includes a similar example for the PCB_20*A*_ pigment, since DBV and PCB are the primary pigment types analyzed in this study. The observed similarity in the spatial distribution of the HOMO and LUMO orbitals but also the difference and transition densities indicate that the electronic transition from the HOMO to the LUMO involves only minimal reorganization of the electron density. Consistently, Table 2 shows that the dipole moments of the ground and excited states only differ slightly. This further supports that both states respond in a similar manner to external electrostatic fluctuations. As a result, fluctuations of point charges in the environment do affect the ground and excited state energies in a similar manner. This insensitivity of the energy gap between excited and ground state results also in an insensitivity of the respective spectral densities to fluctuations in the environment. This interesting feature of bilin pigments was observed earlier in the PE545 complex.^16,17^ Moreover, as detailed in that study, the spatial localization of the HOMO and LUMO orbitals in bacteriochlorophyll molecules is different. Therefore, these orbitals are differently influenced by external charge fluctuations leading to modifications in the energy gap.

**Figure 8:**
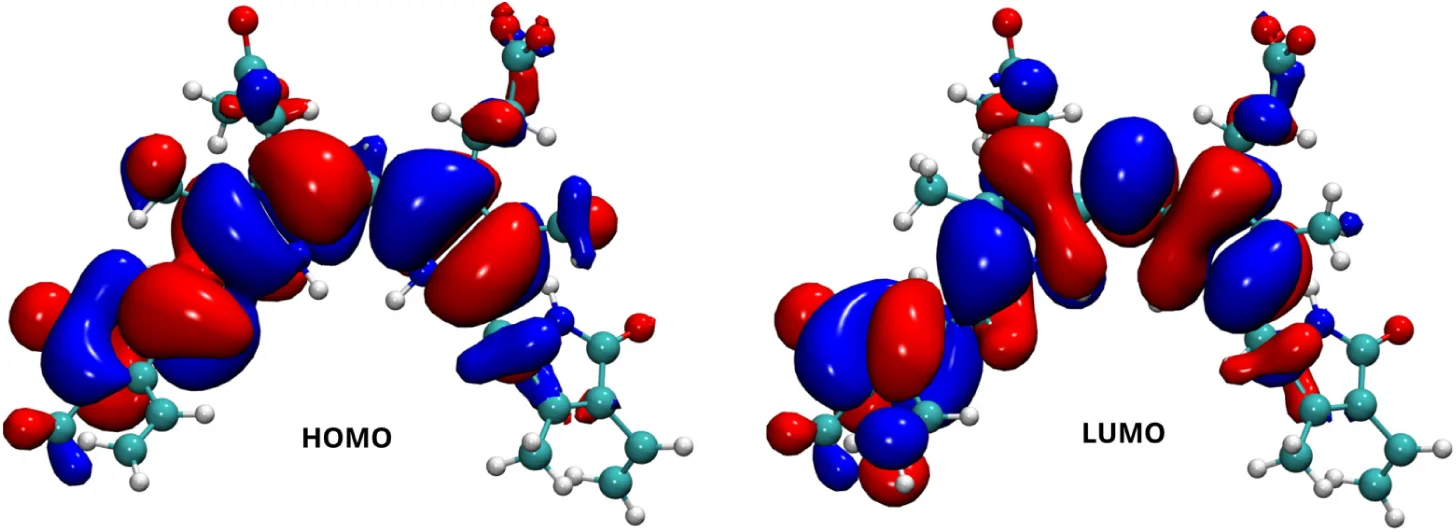
HOMO and LUMO of bilin DBV_50/61*B*_ within QM/MM setting. The isosurface value ranges from +1 × 10^–2^ (blue) to −1 × 10^–2^ (red).

**Table 2:** Average Magnitude of Ground (*S*_0_) and First Excited State (*S*_1_) Dipole Moments *μ* within the Protein Environment and Their Absolute Differences (In Debye)

Bilins	μ(*S*_0_)	μ(*S*_1_)	|Δμ|
DBV50/61*B*	69.25	69.07	0.18
PCB_20*A*_	71.27	73.42	2.15

### Spectral Density Comparison of Open and Closed PBPs

Figure 9 presents the spectral density averaged over all eight pigments, obtained from three independent sets of 40 ps QM/MM MD trajectories. The corresponding results for each individual set are provided in Figure S9 of the Supporting Information. These results are compared to those from the closed-form PC645 complex.^23^ For the latter protein two protonation pattern have been established: one where all eight bilins are protonated and another where two MBV pigments are deprotonated based on their interactions with water molecules at the central pyrrole ring. These two configurations are referred to as the protonated and deprotonated complexes, respectively. Our recent findings indicate that the deprotonated MBV pigment exhibits increased flexibility, resulting in a greater number of high-frequency vibrational peaks. One has to keep in mind that the effect of the changes in the MBV spectral density are averaged out to some extend since the spectral densities of the other pigments in PC645 are unchanged by this change in protonation state. Figure 9 compares the spectral densities of PC612 and both protonation forms of PC645.^23^ Notably, PC612 and the protonated form of PC645 display similar spectral line-shapes at both low and high frequencies, likely due to all pigments being protonated in both systems. At the same time it is very reassuring that the spectral densities of these two different complexes with almost the same pigments are very similar, pointing to a robustness of our employed procedure also concerning sampling. In contrast, the deprotonated PC645 complex shows comparable features in the low-frequency region, but significantly more peaks in the high-frequency region. This increase arises from the enhanced flexibility of the deprotonated MBV, which contributes additional intramolecular vibrational modes.^23^

**Figure 9:**
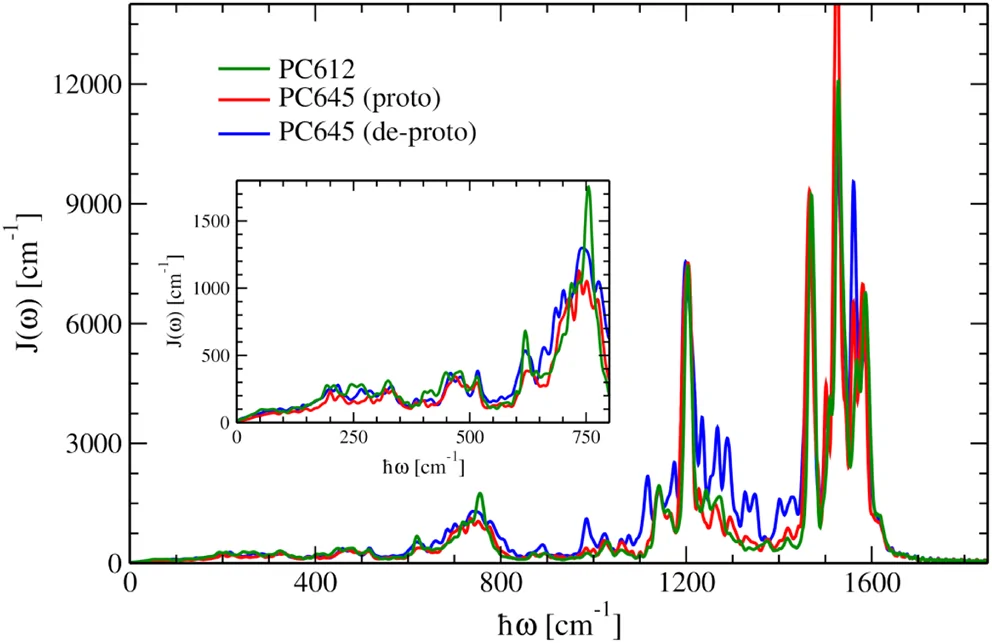
Comparison between the spectral density averaged over all eight pigments based on three 40 ps QM/MM MD trajectories in the PC612 and PC645 complexes.^23^ The inset focuses on the low-frequency region.

Furthermore, it is also important to note that the spectral density intensities for bilins pigments obtained in the calculations are significantly higher than those typically observed in chlorophyll (Chl)^32–35^ and bacteriochlorophyll (BChl) systems.^31^ This difference can be attributed to the intrinsically greater conformational flexibility of bilin molecules. As open-chain tetrapyrroles with conjugated polyene segments, bilins undergo larger structural changes in response to thermal fluctuations than the more rigid porphyrin rings found in Chls and BChls. These structural fluctuations strongly modulate the electronic excitation energies and couple efficiently to C=C and C–C stretching vibrations, resulting in larger reorganization energies. Such large reorganization energies can impact the accuracy of absorption spectra predictions, especially since most theoretical models for absorption spectra assume relatively small reorganization energies.

### Reorganization Energies and Transition Dipole Moments

The reorganization energies λ_*m*_ and transition dipole moments **μ** of the individual pigments are important parameters for modeling the excited states and absorption spectrum of the protein-pigment complex. The reorganization energy λ_*m*_ for pigment *m* can be determined from its spectral density using

6 $$\lambda_{m}=\int_{0}^{\infty }\frac{J_{m}\left(\omega \right)}{\omega }d\omega$$

The results for the pigments in PC612 are listed in Table 3, and the average reorganization energy of the eight bilins is 1643 cm^–1^. The average transition dipole moments were extracted directly from the TD-LC-DFTB calculations performed along the 1 ns QM/MM MD trajectory. The three-dimensional transition dipole moment vectors were averaged by treating the direction and magnitude separately. To this end, we first visualized the calculated transition dipole moments for the first frame of all eight bilin trajectories using VMD^64^ ensuring a consistent orientation. The direction was defined from the protonated, positively charged nitrogen atom in the second pyrrole ring to the nitrogen atom in the third pyrrole ring. Since the sign of the transition dipole moment is not defined, we then aligned the dipole moments across all frames by ensuring that the dot product with the reference of the first frame remained positive. Once properly aligned, we computed the average vector by summing all dipole moment vectors and normalizing their direction. After that, the magnitude was determined by averaging the norm of the individual dipole moments across all frames. Finally, the average magnitude and direction were combined to obtain the mean transition dipole moment. The average magnitudes of all eight bilins reported by Curutchet and coworkers range from 12.6 to 14.8 D^19,27^ based on QM/MMPol calculations using classical MD and *ab initio* QM/MM trajectories. Our results fall within the same range but show slightly smaller values. Since both studies employ QM/MM trajectories, the observed differences can be attributed to the use of the TD-DFTB approach for the excited-state calculations instead of DFT-based methods.

**Table 3:** Reorganization Energies *λ_m_* for the Individual Pigments, Obtained from the Spectral Densities Shown in Figure 7, Along with the Magnitudes of the Average Transition Dipole Moments (*μ*) Determined from 1 ns QM/MM MD Simulations Using the TD-LC-DFTB Method

Bilins	λ_*m*_ (cm^–1^)	μ (Debye)
DBV50/61*B*	1696	11.81
DBV50/61*D*	1658	11.69
PCB_20A_	1785	11.19
PCB_20*C*_	1854	12.75
PCB_158*B*_	1578	12.91
PCB_158*D*_	1637	12.55
PCB_82*B*_	1529	13.16
PCB_82*D*_	1410	13.17

### Absorption Spectra

To validate the properties computed in this study, we modeled the absorption spectra and compared them with experimental measurements. The circular dichroism (CD) spectrum was also calculated and is provided in the SI. Since all calculated spectra were shifted to align with the experimental results, our analysis focuses primarily on the peak separation and the relative peak intensities. Time-averaged system Hamiltonian (using constant screening factor for the excitonic couplings), spectral densities (SD) of the individual bilins and the respective transition dipole moments were employed as input to calculate the absorption spectra of the PC612 complex using the full cumulant expansion (FCE) formalism.^28,29^ The results at temperatures of 295 K and at 77 K are shown in Figure 10. Moreover, we added a stick spectrum based on the positions of the excitonic energies with heights proportional to the square of the excitonic transition dipole moments. The absorption spectrum of the PC612 complex at room temperature presents two distinct peaks,^15^ with a separation of about 1050 cm^–1^. The peak at lower energy is predominantly influenced by the PCBs, whereas the higher-energy peak arises from excitonic states dominated by the DBVs, together with contributions from the vibronic sidebands of the PCBs, as shown in Figure S12 in the Supporting Information. The FCE method employed for the calculation of the absorption spectra includes non-Markovian and nonsecular effects which are neglected, e.g., in Redfield theory. The distance between the two computed peaks is approximately 1200 cm^–1^. This represents a reasonable agreement with experiment, especially considering the improvement over the previous theoretical study of the PC612 complex, which predicted a peak splitting of approximately 1450 cm^–1^.^27^ The relative peak intensities and the overall broadening in the spectrum are still imperfect, mainly due to the tendency of TD-LC-DFTB to overestimate the reorganization energy.^32^ Therefore, the spectral densities were reduced by 30%. This reduction is based on previous studies determining spectral densities using machine-learning for porphyrin molecules attached to clay surfaces^65^ and for BChl pigments in an LH2 complex.^66^ In these studies, excitation and reorganization energies calculated using TD-LC-DFTB and using TD-LC-DFT (CAM-B3LYP/Def2-SVP or Def2-TZVP) were compared. This comparison consistently indicated that the TD-LC-DFTB approach overestimates excitation energies and thus also reorganization energies by roughly 30%. While roughly maintaining the main peak positions, the spectra obtained using the modified spectral densities show an improved agreement with the experimental results in terms of the relative peak intensities and broadenings. A similar tendency can be observed at both temperatures, i.e., 77 and 295 K. The 1230 cm^–1^ peak separation measured experimentally at 77 K is, however, slightly overestimated to be approximately 1335 cm^–1^ with the scaled spectral densities, and notably, the calculated relative peak heights match the experimental finding much better.

**Figure 10:**
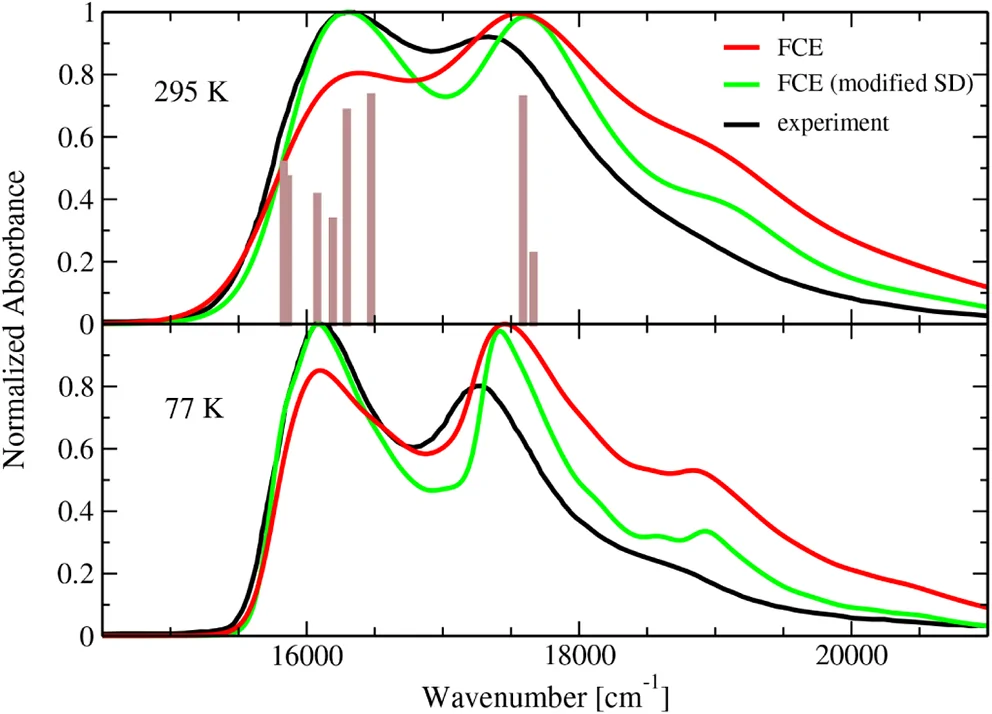
Absorption spectra based on the FCE theory at 295 and 77 K. The results obtained using the original spectral densities are represented in red, and the green curves show the results obtained using a spectral density reduced by 30%. The histogram reveals the excitonic energy peaks. The following shifts have been applied to the calculated results to compare with the experimental spectra:[Bibr ref15] 1850 cm^–1^ (295 K), 2150 cm^–1^ (295 K with modified SD), 1900 cm^–1^ (77 K), 2280 cm^–1^ (77 K with modified SD).

## Conclusions

In this study, we investigated the optical properties of the open-form PC612 antenna complex from cryptophyte algae by using a multiscale strategy. To accurately describe the excited state properties of the pigments while maintaining computational efficiency, we applied QM/MM MD simulations. The dynamics of individual chromophores were treated using the numerically efficient DFTB method, whereas the rest of the system was treated using a classical force field. The resulting site energies, spectral densities, and excitonic couplings collectively enabled a reasonable reproduction of the experimental absorption spectra, highlighting the reliability of the present multiscale framework.

In addition to reproducing the main features of the experimental absorption spectra, the present results also provide a deeper insight into the underlying energy landscape of PC612. The calculated excitation-energy distribution reveals a clear energetic gradient that facilitates directional energy transfer from the high-energy DBV pigments toward the lower-energy PCB_82_ chromophores. This work, together with our previous study on the PC645 complex,^23^ establishes a unified framework for understanding structure–function relationships in cryptophyte antenna systems. Although PC612 and PC645 share highly similar amino acid sequences, subtle differences lead to two distinct conformational states (open and closed forms), which result in significant variations in their energy landscapes and excitonic properties.

An important finding, which was already reported for the PE545 complex^16,17^ but is worth reiterating is the extremely small influence of protein and water fluctuations on the spectral densities. Spectral densities describe how system couple to thermal environments and are ultimately responsible for decoherence and relaxation. Since long-lived coherences were experimentally observed for this kind of systems^21,67^ the present finding might warrant more attention since they are possibly key for a clear microscopic understanding.

The methodology used in the present study can be readily applied to investigate EET processes in other LH complexes, as already demonstrated in previous studies.^23,32–35^ In addition, the excited-state properties obtained in this work provide a valuable foundation for further studies of related cryptophyte antenna complexes, including exciton dynamics and transient absorption spectroscopy. Looking forward, the integration of higher-level excited state methods^66,68^ and nonadiabatic dynamics simulations^69,70^ is expected to provide deeper insight into how biological environments optimize energy transport with remarkable efficiency. Such understanding may also offer useful design principles for the development of artificial light-harvesting systems and bioinspired optoelectronic materials.

## Supplementary Material



## Data Availability

Computational data are publicly available on Zenodo (10.5281/zenodo.20291195), including system equilibration files, QM/MM and classical MD trajectories, as well as site (excitation) energies, spectral densities, and TrESP charges of pigment molecules.

## Notes

See the Supporting Information for further details on the excitation energy ladder, spectral density, and excitation profile of the PCB pigments (PDF)
