# Supplementary material for: Structural Dynamics and Optical Spectra of the Phycobiliprotein PC612 Complex
Source: J Phys Chem B. 2026 Sep 10;130(38):9792–804. doi: 10.1021/acs.jpcb.6c03535 (PMC13618033; doi:10.1021/acs.jpcb.6c03535)
Supplement: Supplementary file 1 [file jp6c03535_si_001.pdf]

Supporting Information for Structural Dynamics and Optical Spectra of the Phycobiliprotein PC612 Complex

Dongyu Lyu[§,†] Sayan Maity[§,†,‡] and Ulrich Kleinekathöfer[*,†]

†*School of Science, Constructor University, Campus Ring 1, 28759 Bremen, Germany*

‡*Department of Physics and Astronomy and Thomas Young Centre, University College London, London, London WC1E 6BT, U.K.*

E-mail: ukleinekathoefer@constructor.university

[§] DL and SM contributed equally to this work.

Determination of the Protonation State

The protonation state of the bilins can significantly affect their excited-state properties. To determine the protonation states of the bilins and their coordinating protein residues, we selected $DBV_{50/61B}$ and PCB_{82B} as representative examples and performed QM/MM MD simulations. Compared to the simulation protocol described in the main text, the QM region in these simulations included not only the bilin molecule but also the side chain of the interacting amino acid residue. Consequently, the protonation states were determined using QM/MM electronic structure calculation, instead of being fixed using empirical or classical electrostatics approaches.

In the initial structures, the proton was placed on the aspartate residue interacting with the bilin, as illustrated in Fig. S1A. Figure S2 shows the distances between the proton and

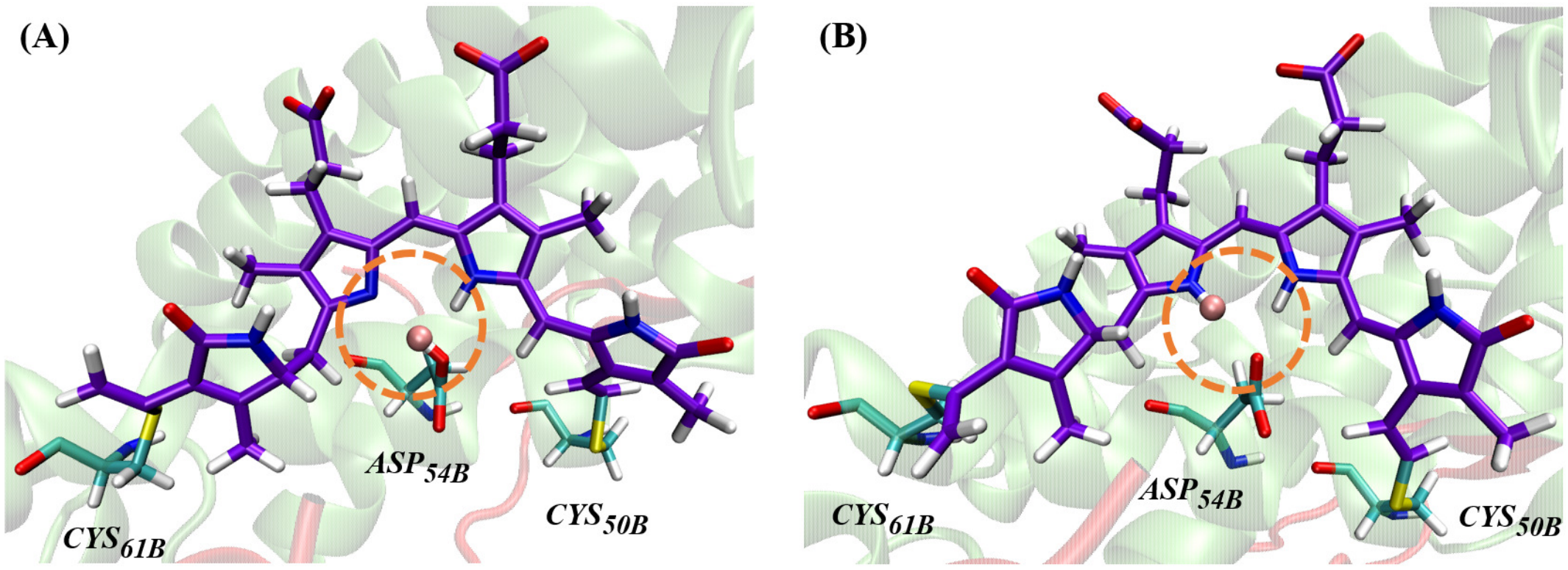

Figure S1: Example of $DBV_{50/61B}$ and its interacting aspartate residue. (A) Initial structure used for the QM/MM MD simulations, in which the interacting aspartate residue was protonated. (B) Final structure after the simulations. The proton (shown as a pink sphere) transferred from the residue to the central pyrrole ring of the bilin.

the protein residue, as well as between the proton and the nitrogen atoms of the central pyrrole rings of the bilin. Within approximately 0.2 ps, the proton was transferred to a nitrogen atom of the central pyrrole rings, as illustrated in Fig. S1B, and it remained stably bound to the bilin throughout the remainder of the simulations without transferring back to the aspartate residue. Therefore, in the simulations presented in the main text and the subsequent excited-state calculations, the central pyrrole rings of the bilins were kept protonated, while the coordinating residues were treated in their deprotonated states.

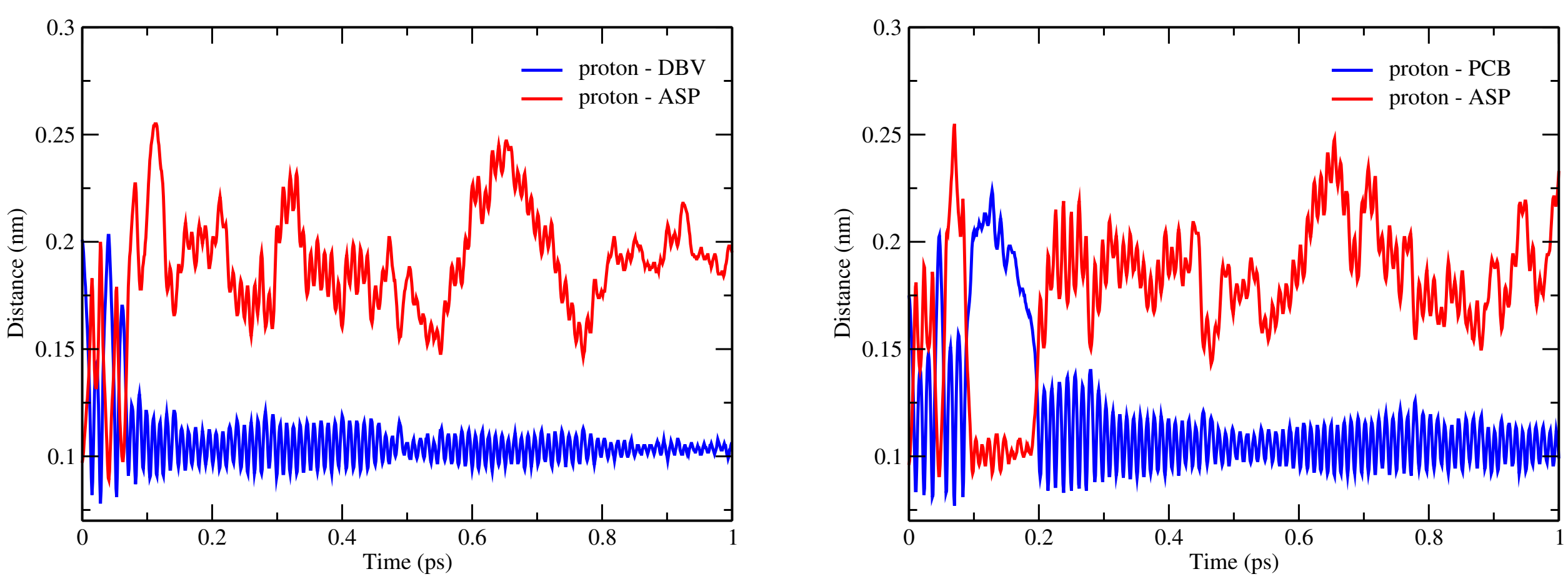

Figure S2: Distances between the proton and the central pyrrole rings of the bilins, as well as between the proton and the interacting aspartate residues. $DBV_{50/61B}$ and PCB_{82B} are shown in the left and right panels, respectively.

Computational Protocols

Fig. S3 lists the simulation protocol employed to generate each trajectory and summarizes the subsequent analyses and calculations performed using the corresponding trajectory.

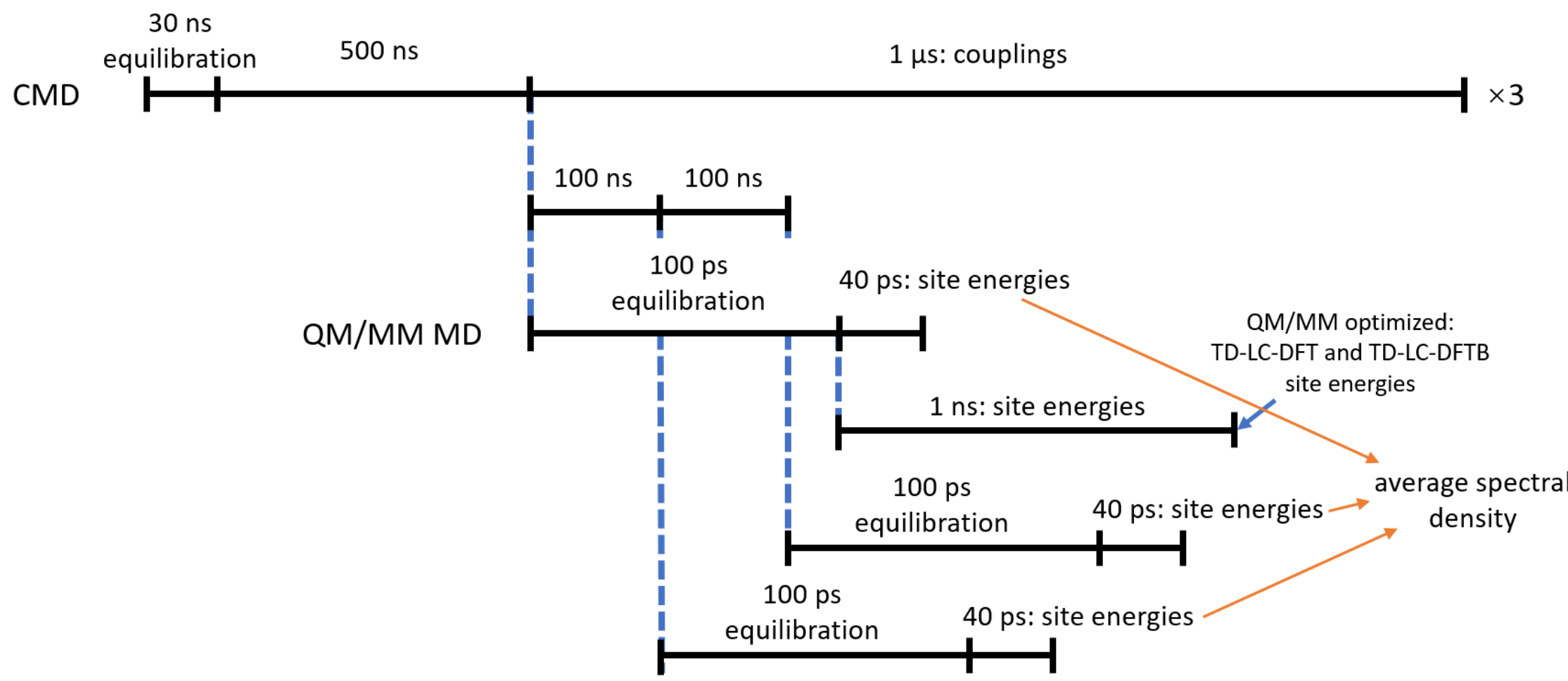

Figure S3: Overview of the trajectories used in this study, including both classical MD (CMD) and QM/MM MD trajectories.

Site Energies

The site energy values of the bilin molecules in the PC612 complex were obtained using several different methods. Initially, TD-LC-DFT (CAM-B3LYP/Def2-TZVP) excited state calculations were performed on the QM/MM optimized structures to serve as a benchmark.

Table S1: Site energies with different methods in units of eV.

	TD-LC-DFT	TD-LC-DFTB		
	QM/MM opt	QM/MM opt	40 ps QM/MM MD	1 ns QM/MM MD
$DBV_{50/61B}$	2.853	2.683	2.552	2.570
$DBV_{50/61D}$	2.764	2.595	2.582	2.578
PCB_{20A}	2.578	2.457	2.402	2.405
PCB_{20C}	2.578	2.469	2.435	2.424
PCB_{158B}	2.577	2.475	2.390	2.387
PCB_{158D}	2.515	2.384	2.412	2.405
PCB_{82B}	2.502	2.352	2.361	2.353
PCB_{82D}	2.538	2.372	2.360	2.357

Subsequently, TD-LC-DFTB calculations were carried out on the same structures. The consistent trend in site energies observed from both methods supports the reliability of TD-LC-DFTB for this system, which was then applied to the 40 ps and 1 ns QM/MM MD trajectories, yielding highly consistent average energy values. The numerical values of the excitation energies for each pigment, as illustrated in Fig. S4, are presented in Table S1. And the distributions of the excitation energies obtained along the 1 ns QM/MM MD simulation are shown in Fig. S5. The system displays a notable degree of symmetry: regardless of the chromophore type, the bilins bound to the same residue site in chain B and in chain D (or chain A and C) exhibit not only quite similar site energies but also very comparable energy distributions.

As mentioned in the main text, it can be challenging to accurately identify the target state of the bilin molecules due to the relatively small energy gaps between the low-energy excited states. In the present study, we examined the five lowest excited states and selected

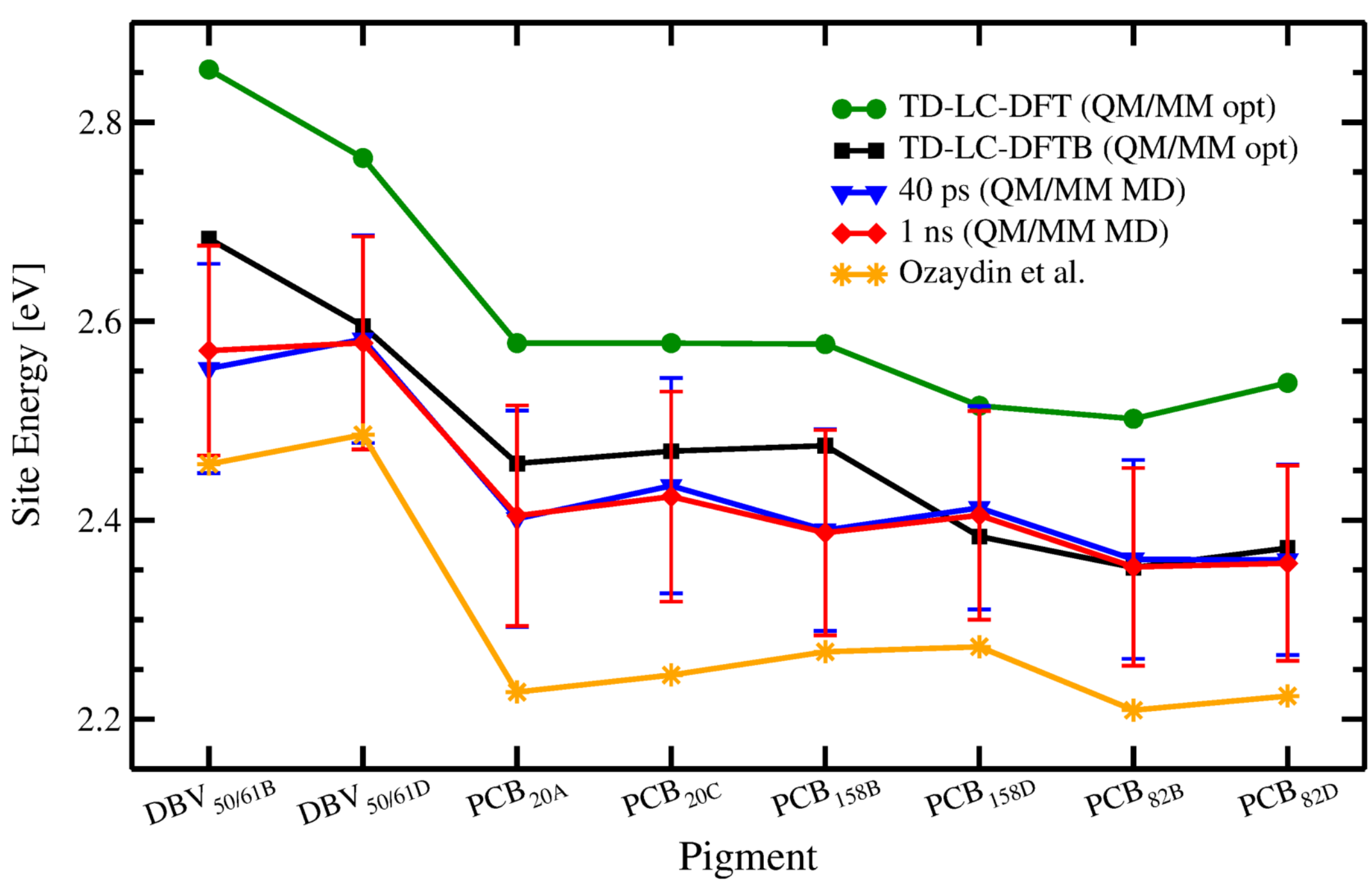

Figure S4: Raw site energies of the bilins in the PC612 complex obtained in the present work and reported by Ozaydin et al.[1], shown without applying any energy shift. The error bars represent the standard deviations of the site energies along the trajectories, reflecting the magnitude of energy fluctuations rather than the uncertainty of the calculations.

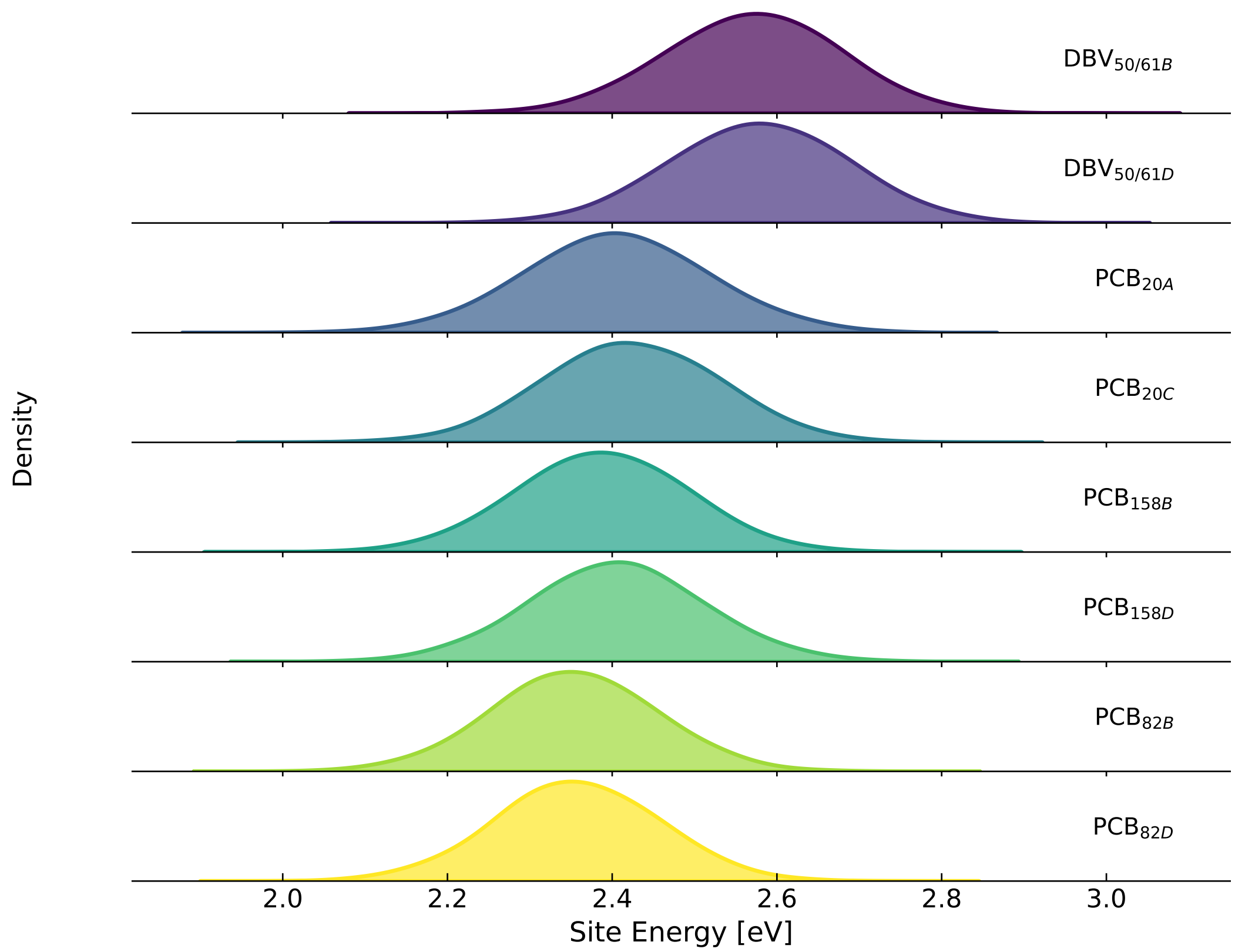

Figure S5: Site energy distributions of the bilin molecules embedded in the PC612 complex based on the TD-LC-DFTB approach along the 1 ns-long QM/MM MD trajectory.

the one with the largest transition dipole moment strength as the target state, from which the site energy was extracted. Figure S6 presents an example segment from the QM/MM MD trajectory of the $DBV_{50/61B}$ molecule, illustrating the excitation energies of the first three excited states obtained using the TD-LC-DFTB method. The extracted target state, determined based on the transition dipole moment, is indicated by the dashed line. In most frames, the first excited state overlaps with the target state. In some cases, however, the second or third excited state is selected. In rare instances, even higher excited states are identified along the trajectory. During state extraction for environments with fixed point charges along the trajectory, many frames showed artifacts for the energy of the lowest excited states. To accurately identify the correct state, it was necessary to consider in addition the dipole moment and/or oscillator strength, as negatively charged bilin pigments are known to exhibit mixing between the lowest and higher excited states[2,3].

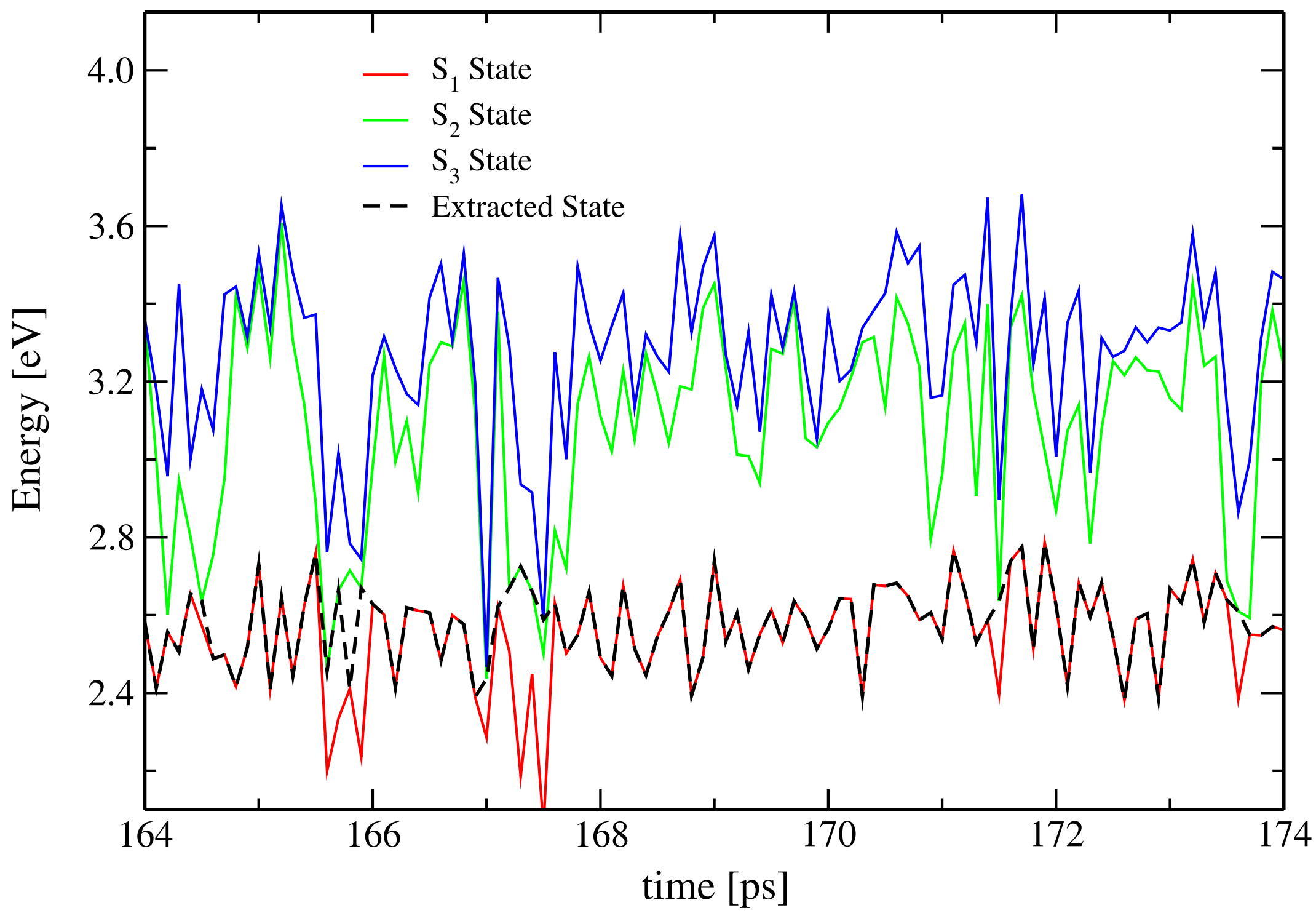

Figure S6: An example segment of an excitation energy time series. The selected excited state is extracted based on the transition dipole moment strength. This time series fragment is taken from excited state calculations performed along the 1 ns QM/MM MD trajectory of the $DBV_{50/61B}$ molecule. The dashed line indicates the selected state, which in most cases corresponds to the first excited state, although for certain frames, the second or even higher excited states were extracted due to level crossing.

Based on the excitonic Hamiltonian constructed using couplings obtained with a constant screening factor as described in the main text (the upper-right part of Table 1), we diagonalized the matrix. The resulting contributions of the individual sites to the excitonic states are listed in Table S2.

Table S2: Contributions (in percentage) of the individual pigments to the excitonic states in the PC612 complex.

	**Exciton 1**	**Exciton 2**	**Exciton 3**	**Exciton 4**	**Exciton 5**	**Exciton 6**	**Exciton 7**	**Exciton 8**
$DBV_{50/61B}$	0.14	0.00	0.01	0.00	0.06	0.09	90.57	9.21
$DBV_{50/61D}$	0.03	0.12	0.00	0.02	0.01	0.07	9.13	90.62
PCB_{20A}	0.05	0.65	17.56	0.55	81.16	0.00	0.03	0.00
PCB_{20C}	0.20	0.10	0.01	22.56	0.21	76.87	0.02	0.02
PCB_{158B}	0.34	0.01	82.11	0.04	17.42	0.03	0.05	0.00
PCB_{158D}	0.00	0.17	0.04	76.74	0.36	22.62	0.01	0.06
PCB_{82B}	97.63	1.53	0.16	0.06	0.28	0.20	0.14	0.00
PCB_{82D}	1.60	97.42	0.11	0.03	0.50	0.19	0.05	0.09

Table S3: Differences in site energies when the QM region includes only the bilin molecule and when it includes both the bilin and the side chain of the interacting residue.

	$DBV_{50/61B}$	PCB_{82B}
site energy with original QM region (eV)	2.570	2.353
site energy with enlarged QM region (eV)	2.563	2.356
difference (eV)	0.007	0.003

The protonation state of bilins has a significant influence on their excited-state properties, while the protonation state itself is determined by the coordinating amino acid residue. In the calculations presented in the main text, the QM region included only the bilin molecule, whereas the interacting amino acid residue was described by classical force-field point charges. To assess the reliability of this QM/MM partitioning scheme, we selected one representative DBV and one PCB to perform additional 40 ps QM/MM MD simulations using an enlarged QM region that included both the bilin and the side chain of its interacting amino acid. A total of 40,000 snapshots extracted from the trajectories were subsequently used to calculate the excitation energies. The resulting average excitation energies are summarized in Table S3. They are in good agreement with those reported in the main text, indicating that including the interacting amino acid residue in the QM region has only a minor effect on the calculated excitation energies.

Excitonic Couplings

Figure S7 presents the distributions of excitonic couplings obtained from three independent 1 μs MD trajectories. To characterize the conformational states of PCB_{20A} in the corresponding trajectories, we calculated the distance between the nitrogen atom of ring A in PCB_{20A} and the carboxyl group of the Glu16A side chain, which is shown in the lower panels of Fig. S7. When this distance is larger than 0.4 nm, ring A of the bilin remains in a conformation similar to that in the initial crystal structure, without undergoing the flipping motion. In contrast, a distance of approximately 0.25 nm indicates that ring A has flipped

to the other side of the molecule.

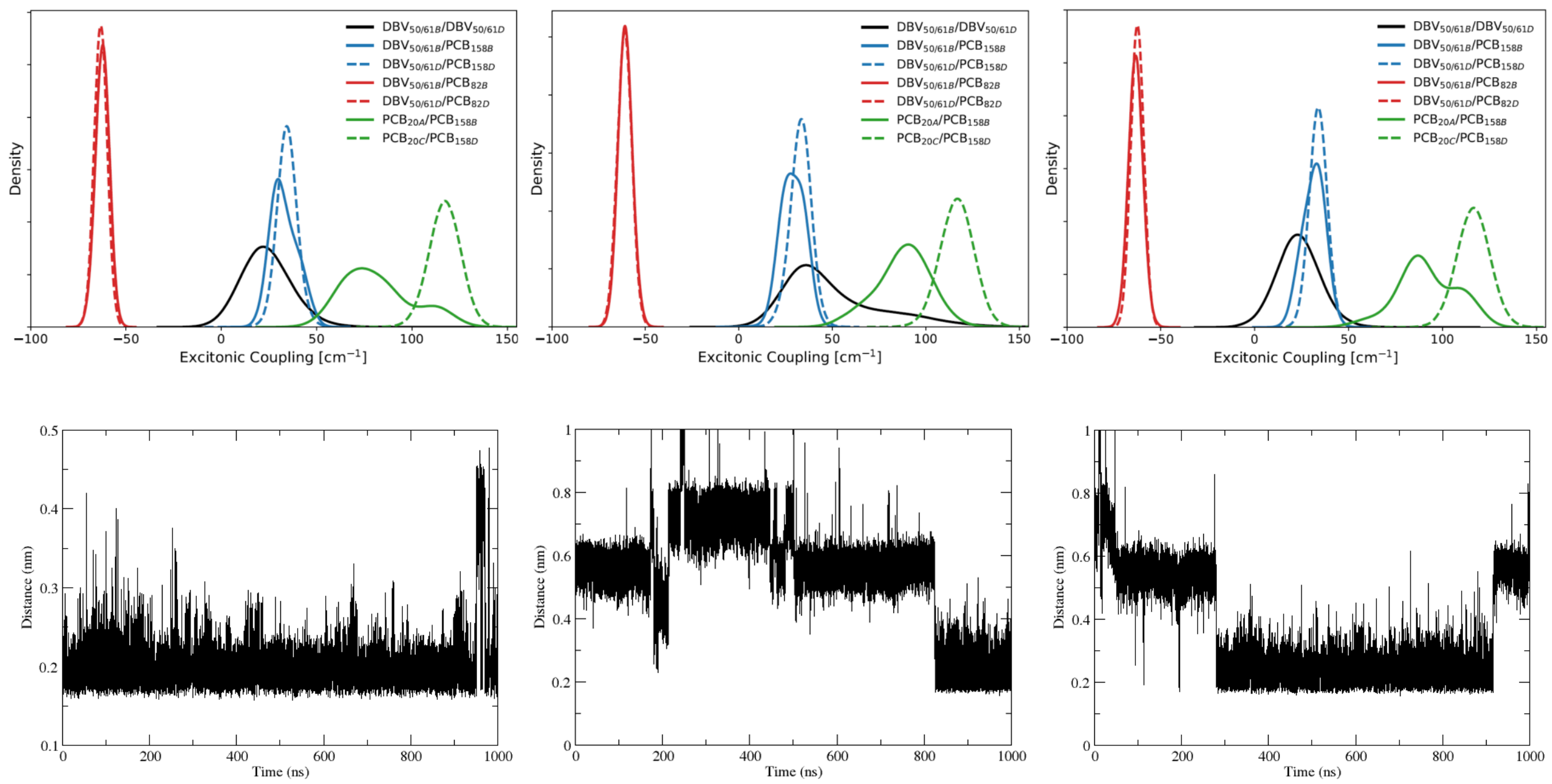

Figure S7: Distributions of the excitonic couplings obtained from three different 1 μs MD trajectories, and the distance between the nitrogen atom of ring A in PCB$_{20A}$ and the carboxyl group of the Glu16A side chain.

Spectral Densities

In the main text, we compared the low-frequency region of the spectral densities obtained under two conditions: with fluctuating (flexible) environmental point charges and with fixed (frozen) point charges. The complete spectral densities of the individual bilins in PC612 complex are depicted in Fig. S8. Since the high-frequency region corresponds to intramolecular vibrational modes, it is relatively insensitive to changes in environmental fluctuations. The peak positions in the spectral densities remain the same, with only slight variations in intensities.

We selected three different initial structures from a 200 ns MD trajectory at 100 ns stride for QM/MM MD simulations and averaged the resulting spectral densities for subsequent analysis. Fig. S9 compares the spectral densities obtained from the three individual sim-

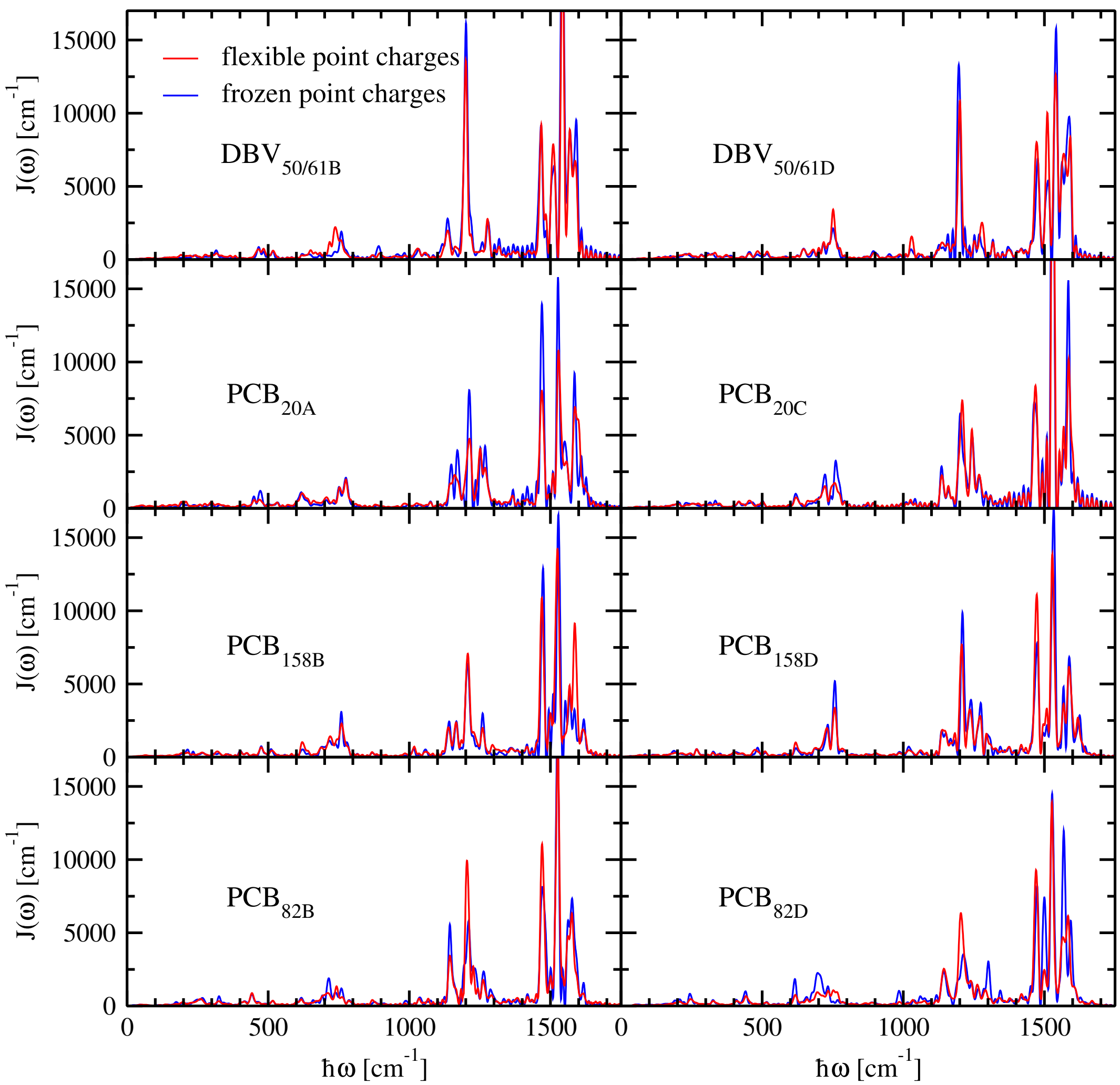

Figure S8: Spectral densities of the eight individual bilins in the PC612 complex based on the same QM/MM MD trajectories with flexible and fixed environmental point charges.

ulations. Although the peak positions in the high-frequency region remain the same, the peak heights and the low-frequency area show significant differences due to sampling variations. This was observed earlier in bacterial or plant light-harvesting complexes[4,5], where we found that the initial excitation of internal pigment modes and the subsequent energy redistribution within the molecules occur more slowly in QM/MM MD simulations.

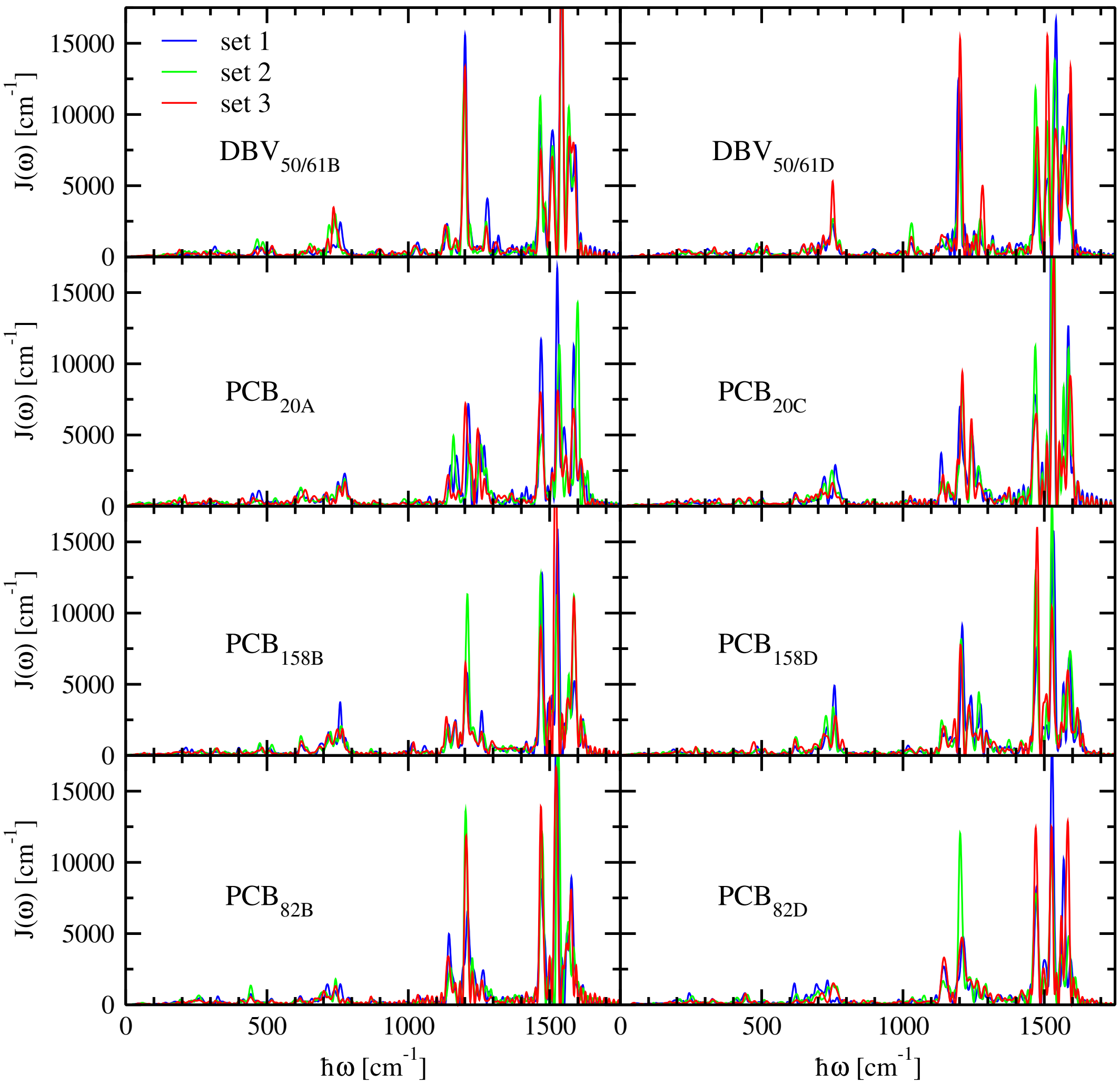

Figure S9: Difference between the spectral densities of individual bilins in the PC612 complex based on three 40 ps QM/MM MD trajectories.

Excitation Profile of Bilin Pigment within Protein

Similar to the DBV pigments discussed in the main text, we have also extracted the excitation profile of the PCB bilin, as these are the two types of pigments present in the PC612 complex. Specifically, we obtained the HOMO and LUMO of the PCB$_{20A}$ pigment within the QM/MM framework, as shown in Fig. S10. The spatial distributions of all excited-state properties are similar, which primarily explains the minimal change in spectral density when using a flexible point charge environment, compared to the case where point charges are kept frozen, as illustrated in Fig. S8. As mentioned in the main text, this unique property of bilin

pigments was seen earlier in the PE545 complex, unlike the bacteriochlorophyll pigments in the FMO complex[2].

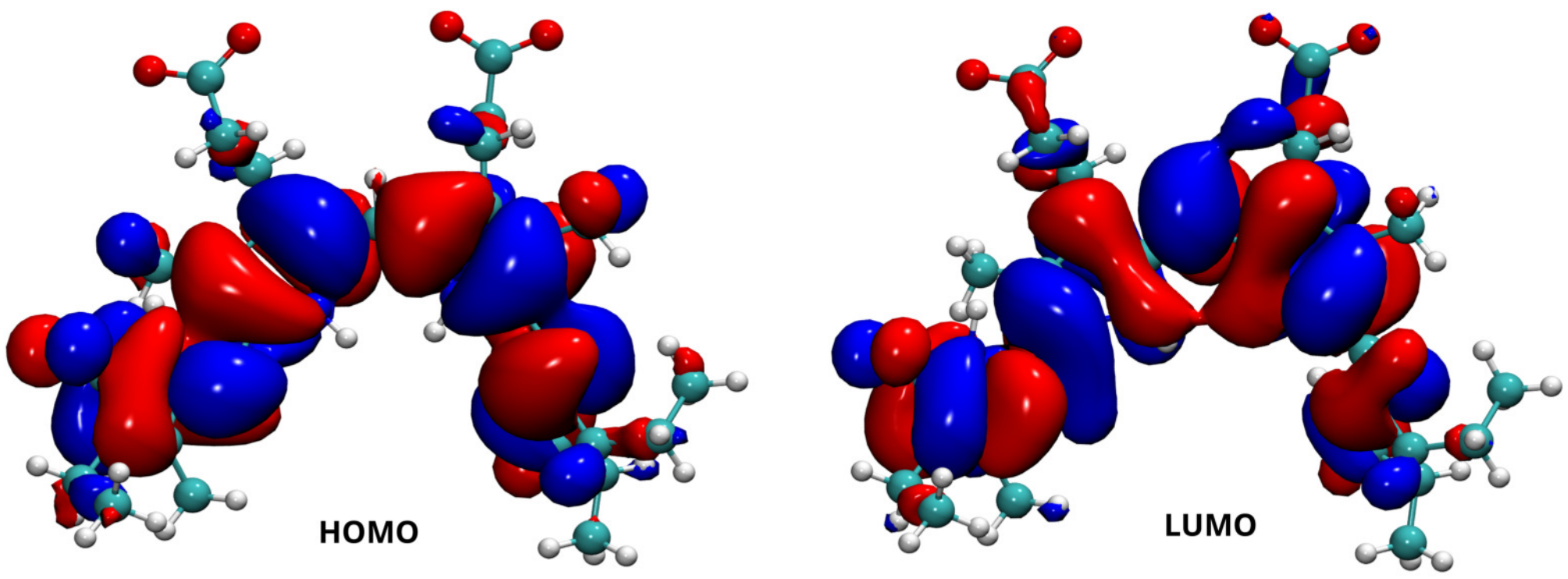

Figure S10: HOMO and LUMO of bilin PCB_{20A} within QM/MM setting.

Absorption Spectra

Since we find that the external electric field (i.e., the surrounding environment) has only a limited influence on the excitation energy fluctuations of the bilins, it is also possible that over a sufficiently long timescale, all PCBs may eventually converge to similar excitation energies. However, verifying this hypothesis would require substantially longer simulations together with more advanced sampling techniques. Nonetheless, if we assume identical average site

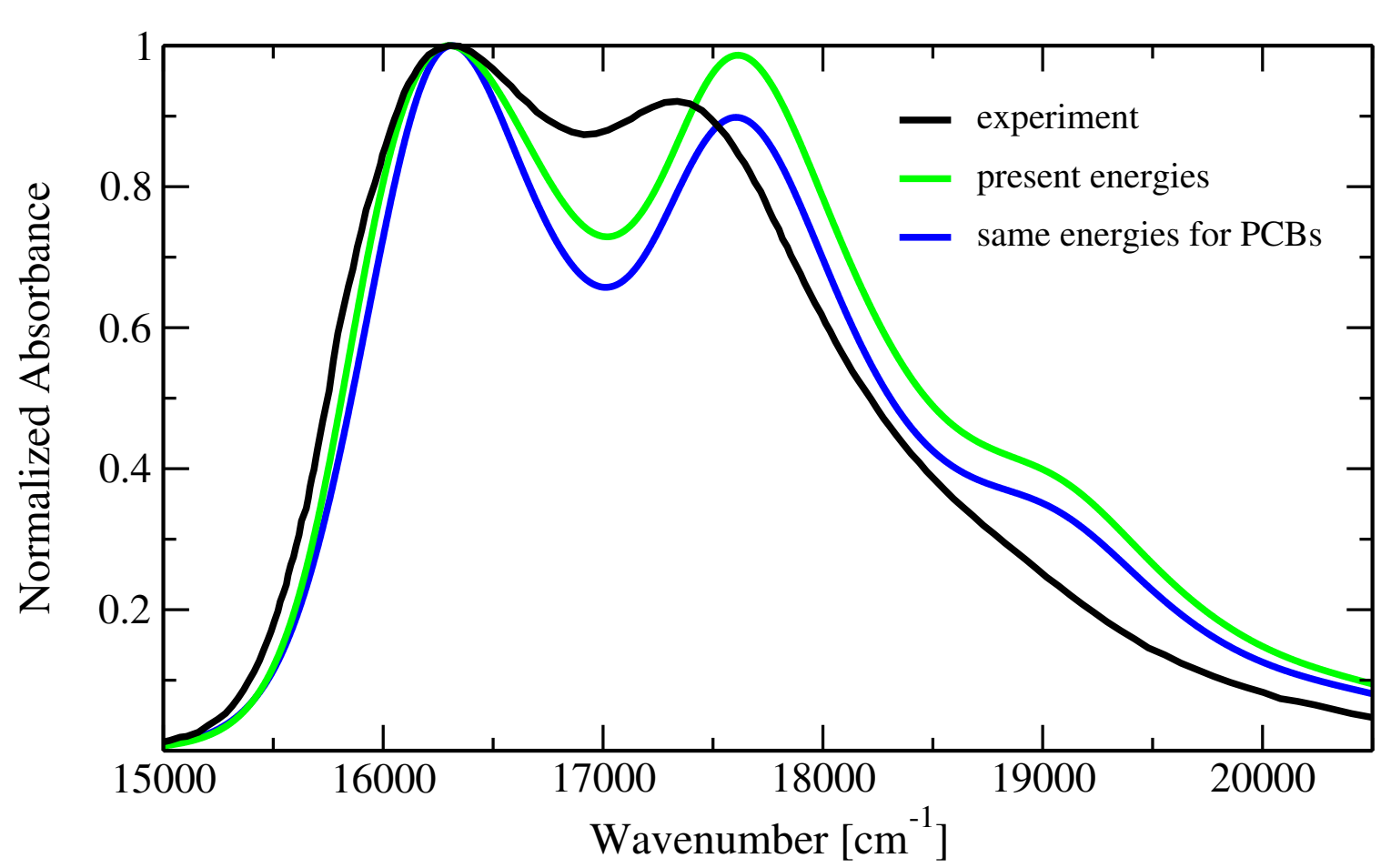

Figure S11: Absorption spectrum of PC612 calculated using three different excitonic coupling datasets.

energies for all PCBs, the intensities of the absorption peaks shows a slightly better agreement with the experimental results, but the peak positions are almost identical, as shown in Fig. S11.

To further investigate the detailed contributions to the individual absorption bands, we calculated the absorption spectra of the systems containing only the PCB and only the DBV pigments at 295 K using the same approach as described in the main text. As shown in Fig. S12, the lower-energy peak is exclusively contributed by excitonic states dominated by the PCB molecules, whereas the higher-energy peak originates from DBV-dominated excitonic states, with additional contributions from the vibronic sidebands of the PCB pigments.

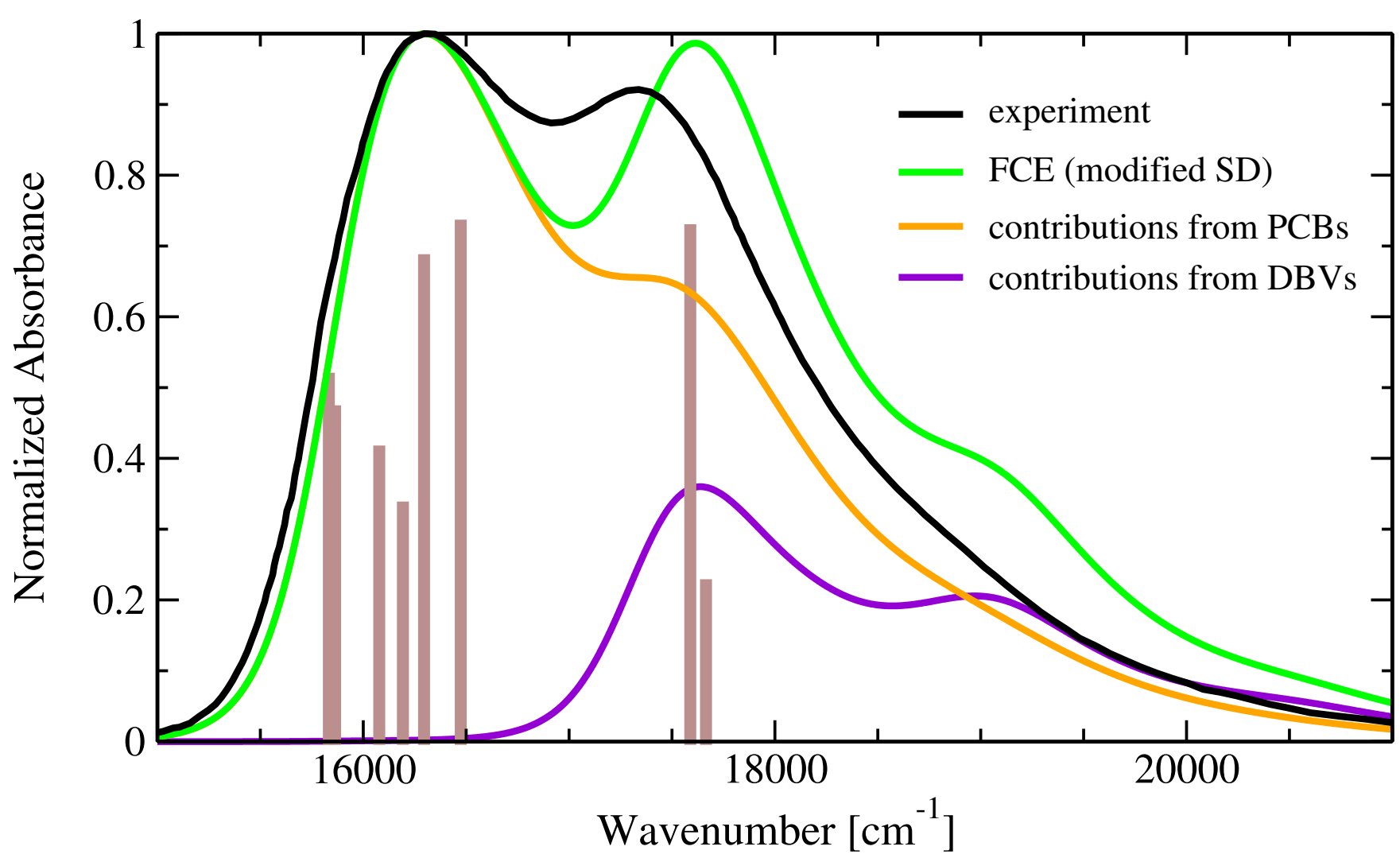

Figure S12: Absorption spectrum of PC612 calculated using the FCE approach at 295 K, together with the individual contributions from the two types of bilin, and the corresponding stick spectrum. All calculated results are shifted by 2150 cm^{-1}.

CD spectra

Also calculated the circular dichroism (CD) spectrum of PC612 has been calculated using the FCE approach. As shown in Fig. S13, the calculated CD spectrum reproduces the positions of the major experimental bands well. Moreover, for a more direct comparison, the theoretical CD spectrum reported by by Ozaydin et al.[1] is included in the figure as well.

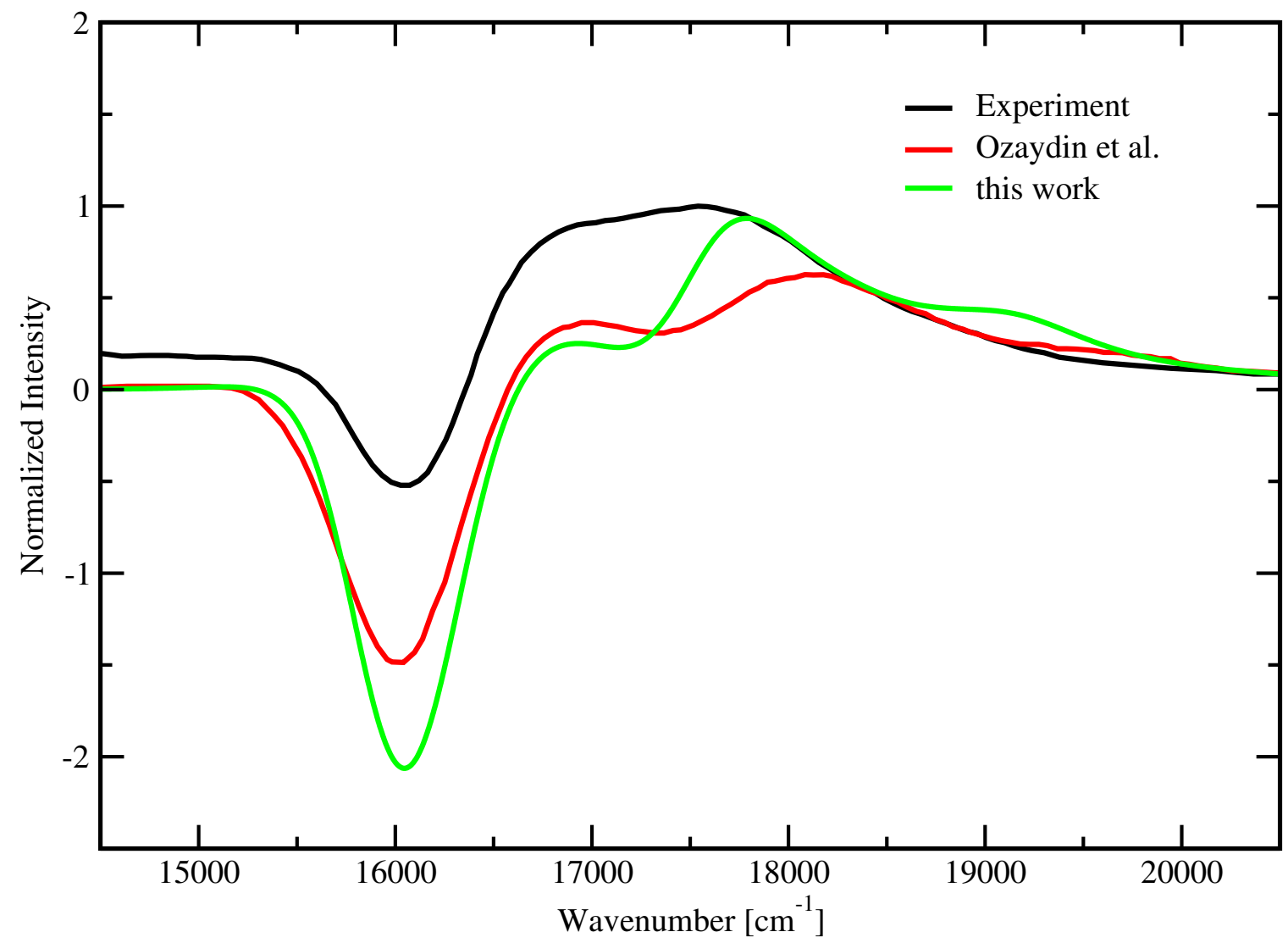

Figure S13: CD spectrum of PC612 calculated at 295 K using the FCE approach, compared with experimental data and previous theoretical results reported by Ozaydin et al. Our result has been shifted by 2185 cm^{-1}.

References

(1) Ozaydin, B.; Curutchet, C. Unraveling the Role of Thermal Fluctuations on the Exciton Structure of the Cryptophyte PC612 and PC645 Photosynthetic Antenna Complexes. *Front. Mol. Biosci.* **2023**, *10*, DOI: 10.3389/fmolb.2023.1268278.

(2) Aghtar, M.; Strümpfer, J.; Olbrich, C.; Schulten, K.; Kleinekathöfer, U. Different Types of Vibrations Interacting with Electronic Excitations in Phycoerythrin 545 and Fenna-Matthews-Olson Antenna Systems. *J. Phys. Chem. Lett.* **2014**, *5*, 3131–3137, DOI: 10.1021/jz501351p.

(3) Aghtar, M.; Kleinekathöfer, U.; Curutchet, C.; Mennucci, B. Impact Of Electronic Fluctuations And Their Description On The Exciton Dynamics In The Light-Harvesting Complex PE545. *J. Phys. Chem. B* **2017**, *121*, 1330–1339, DOI: 10.1021/acs.jpcb.6b10772.

(4) Maity, S.; Bold, B. M.; Prajapati, J. D.; Sokolov, M.; Kubař, T.; Elstner, M.; Kleinekathöfer, U. DFTB/MM Molecular Dynamics Simulations of the FMO Light-

Harvesting Complex. *J. Phys. Chem. Lett.* **2020**, *11*, 8660–8667, DOI: 10.1021/acs.jpclett.0c02526.

(5) Maity, S.; Daskalakis, V.; Elstner, M.; Kleinekathöfer, U. Multiscale QM/MM Molecular Dynamics Simulations of the Trimeric Major Light-Harvesting Complex II. *Phys. Chem. Chem. Phys.* **2021**, *23*, 7407–7417, DOI: 10.1039/D1CP01011E.
